# Intentional policy graphs: A pipeline for explaining agent behavior through intentions

**DOI:** 10.1016/j.patter.2026.101513

**Published:** 2026-04-10

**Authors:** Victor Gimenez-Abalos, Sergio Alvarez-Napagao, Adrian Tormos, Sara Montese, Ulises Cortés, Javier Vázquez-Salceda

**Affiliations:** 1 Barcelona Supercomputing Center, Plaça Eusebi Guell, 1-3, 08034 Barcelona, Spain; 2 Universitat Politecnica de Catalunya, c/Jordi Girona, 1-3, 08034 Barcelona, Spain

**Keywords:** XAI, intentions, post hoc explainability, agent explainability, telic explanations, interpretability, reliability, explainable agency

## Abstract

Agents increasingly operate in complex environments, where coherent behavior often emerges from opaque decision-making processes. While such systems can be highly effective, this lack of transparency limits trust, auditing, and meaningful human understanding. We introduce intentional policy graphs, a post hoc, model-agnostic framework that explains agent behavior in terms of intentions: probabilistic commitments to desired outcomes inferred from partial observations. By extending policy graphs with a formal notion of intention, we move beyond action-level descriptions toward telic explanations of why agents pursue particular trajectories. The framework provides a complete construction pipeline, design principles, and quantitative metrics that explicitly characterize the trade-off between interpretability and reliability. Intentions support structured answers to what, how, and why questions, enabling both local and global explanations of behavior. We demonstrate the approach in a cooperative multi-agent game and on real-world human driving data, highlighting its generality and explanatory power without access to internal reasoning models.

## Highlights

• Explains opaque agent behavior using intentions rather than low-level features
• Introduces intention-based metrics for interpretability and reliability
• Works from partial observations without access to agent internals
• Validated in two use cases: a cooperative game and human driving scenarios

## The bigger picture

Artificial intelligence (AI) agents increasingly operate autonomously in complex environments. While these systems can exhibit highly effective and adaptive behavior, they are often opaque, making it difficult for users, developers, and regulators to understand why agents act as they do. This lack of understanding undermines trust, accountability, and the safe deployment of AI systems. This work contributes to the broader goal of trustworthy AI by introducing a methodology to explain agent behavior in terms that align with how humans naturally reason about actions: through intentions and goals. Rather than focusing only on low-level correlations between states and actions, we model behavior in terms of what an agent is trying to achieve and how strongly it is committed to those objectives. This allows explanations that answer intuitive questions such as what the agent wants to do, how it plans to do it, and why a particular action makes sense in context.

By building these explanations from partial observations and without access to the agent’s internal model, the approach is applicable to opaque or proprietary systems. Furthermore, the proposed metrics make it possible to reason explicitly about the trade-off between interpretability and reliability of explanations. Together, these contributions support better auditing, tracing, observing, debugging, and monitoring of autonomous agents, enabling human understanding of these systems.

## Graphical abstract

**Figure:**
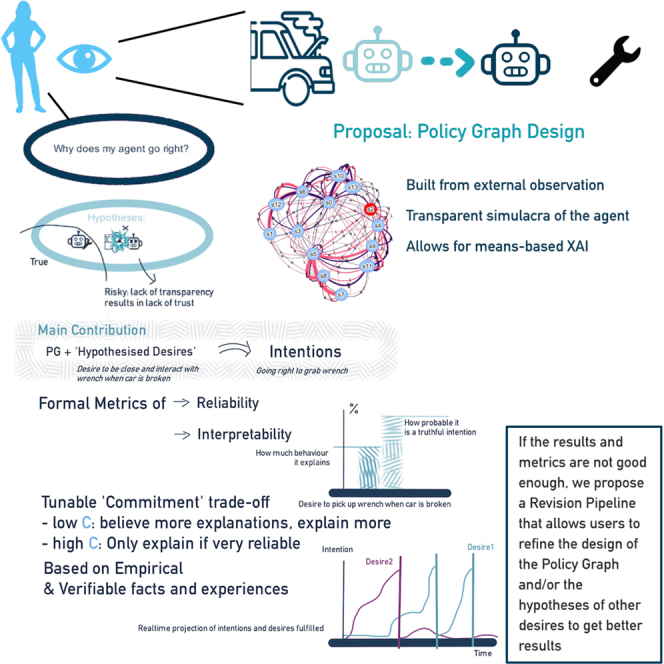

## Teaser

Modern AI agents often act in ways that are hard to understand, even when their behavior is effective. This work introduces a method to explain agent behavior in terms of intentions: (1) what the agent is trying or is committed to achieve and (2) why its actions make sense toward an intended goal or outcome. By relying only on observed behavior, this approach applies to opaque systems and helps support trustworthy AI.

## Introduction

Nowadays, there are lots of efforts to develop artificial intelligence (AI) systems that can solve complex problems without the need to provide them with explicit knowledge on *how* to solve them. This is often achieved through data-driven machine learning (ML) methods, which produce an artifact that, unless explicitly designed to be transparent, is often not interpretable and therefore not trustworthy.^1^^,^^2^ This presents new challenges to the field of explainable AI (XAI).

One of the requirements of trustworthy AI systems is the capability to explain their behavior properly. A model explanation is an exercise in communication between a sender or source (i.e., the model or one of its components) and a receiver (i.e., the explainee, a human or another processor for a downstream task) that describes the relevant context or the causes surrounding some facts,^3^^,^^4^^,^^5^ which in the context of AI is often related to its final or intermediary outputs or decisions. While any communicative act can be considered an explanation, not all explanations are useful or desirable. According to empirical studies,^6^ it can be argued that the form of an explanation should align with its function, as an answer to a question, within a conversational framework.

There are more works that expound on the properties that an explanation should have. Herbert P. Grice^7^ proposed four maxims for useful collaborative communication. One of them (manner) is prevalent in the literature, renamed as interpretability^2^: the explanation should be comprehensible and clear to the receiver. Another (quality) is gaining weight in the community, renamed as reliability^8^^,^^9^^,^^10^: that the explanation contains truthful information. However, interpretability and reliability as two separate objectives will often conflict, requiring careful optimization. For instance, consider a complex ML model. The most reliable explanation would involve a detailed breakdown of its code, while the most interpretable explanation might be a simplified, abstracted, and potentially misleading description of its behavior.

Partly solving this issue is the question of pragmatism: what is explainability used for? Depending on the particular objective of a communicative exercise, this can set which is the right trade-off between interpretability, reliability, and other properties. Some objectives include justifying previous actions of the explainer, controlling and correcting its future behavior, improving the behavior via explainee feedback, and for the explainee to discover knowledge of the explainer to learn from it. As such, any desirable XAI algorithm is tackling at least one of these objectives^2^^,^^4^^,^^11^ while holding some notions (often implicit) of the desirability of explanations related to some of Grice’s maxims.

When explaining models that can be easily accessed, this task is already complex enough. However, with the increase in deployment of opaque models, auditing depends on developers’ willingness to disclose data sources, design principles, and models, as well as to provide auditing tools to the community.^12^^,^^13^ When this is not the case, validating a model as a user becomes unachievable. We, as a community, need better tools to tackle this problem.^14^

This is particularly the case for autonomous agents^15^ that interact in an environment: understanding an agent’s purpose or assumed intentions (the target of the explainable agency [XAg] subfield of XAI) is a hard problem, especially if one has no access to the model or it is opaque. This is even harder when the explainee has no access to its motivation (e.g., the reward function in the case of reinforcement learning [RL], goals and desires for belief-desire-intention [BDI], etc.) or if the agent is not entirely rational (i.e., acting always in accordance with its goals). In these cases, obtaining explanations becomes an exercise in anthropomorphism, where a human interpreter attributes behaviors (based on what a human would do, as shown by Heider and Simmel^16^) in a qualitative analysis that may be inaccurate and risks self-deception and harm.^17^^,^^18^

This issue is compounded by the state of current XAg: in explaining inherently opaque state-of-the-art agents—particularly those trained via RL—traditional explainability techniques typically focus on statistical relationships between inputs and outputs. Please note that by opaque, we mean that the internal policy, reward function, means-ends reasoner, or other decision-making mechanisms of the agent are not accessible, either by design or due to practical constraints. In this context, these explainability techniques fail to capture the underlying reasons behind an agent’s actions. In contrast, the burgeoning field of explanations based on telic (or goal-oriented) reasoning should allow a deeper understanding of why an agent behaves in a particular way, increasing the predictability of its behavior rather than just describing what it does. By modeling agent behavior in terms of long-term objectives and decision-making strategies, we can move beyond mechanistic descriptions and toward explanations that are interpretable and actionable for human explainees. Furthermore, providing such analysis as quantitative, verifiable, and reliable explanations will increase the trustworthiness of AI-based systems by having the *explainee* be aware of the reliability and interpretability of explanations provided and have ways to compare them. Following this rationale, in this paper, our focus is on methodologies for explaining the behavior of unknown agents: agents that are opaque or that have a behavioral policy or model that cannot be inspected. From now on, we assume only partial observations of agent actions and environment states. Additionally, we will assume that we have access to a (potentially incomplete) notion of what the desirable behavior should be in terms of what is needed to control, improve, or justify the actions of the agent,^11^^,^^19^ from an explainee point of view.

The paper builds on the original policy graph (PG) framework,^20^ an explanation model based on probabilities derived from observing an agent (agnostic to its internal functioning, and thus post hoc^11^^,^^21^). This model and its extensions^22^^,^^23^^,^^24^ have been applied to several use cases, but in this paper, we focus on the extension to intentions.^25^ This method requires no access to the agent program or model, instead relying on (potentially partial) observations over actions and states reached by the agent, without needing access to the reward function, internal state, or design criteria, and provides teleological or telic (i.e., goal oriented: explanations in terms of an objective *causing* the action, instead of referring to properties of the state alone). These explanations are generated from desires that are *hypothesized* (but not verified) by the human explainer to belong to the agent. This work extends previous work by doing the following.

**Figure 1:**
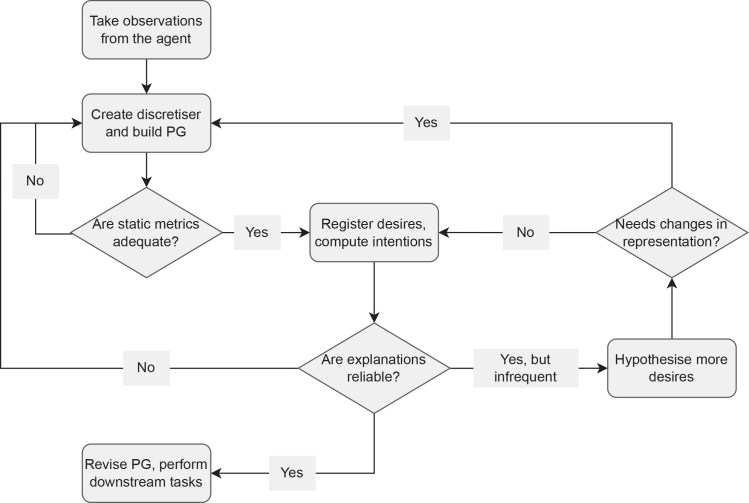
Proposed workflow for extracting explainability First, (partial) observations of the agent interacting in the environment are taken. The system designer (in collaboration with future explainees) can already hypothesize some situations the agent appears to find desirable (i.e., hypothesized desires) based on their own understanding of the environment and/or their observation of the agent. The system designer then proposes a (or several) discretizer(s) to describe the states, following policy graph construction and design heuristics, written in a code comprehensible to downstream explainees that allows them to check or verify which states or transitions satisfy the hypothesized desires. Then, the resulting PG can be evaluated with static metrics, allowing the user to gauge the complexity of the PG representation and loop back to check different representations (e.g., if the discretizer is so complex that the number of observations is insufficient to estimate transition probability or the agent’s policy). When the designer considers the discretizer adequate according to those metrics, the hypothesized desires are formalized and introduced into the PG, which are used to compute intentions. To validate the desires and give direct estimands of reliability and interpretability, a minimum commitment threshold for intention attribution is chosen based on intention metrics, which allow us to evaluate how much of the behavior is explained by attributed intentions and how likely an attributed intention is to be fulfilled and use this information to revise the commitment threshold, revise their hypotheses, employ the revision pipeline to identify which aspects of behavior remain unexplained and may motivate new desires, or debug the agent. The pipeline also allows explainees to use explanation algorithms to answer queries about agent behavior.
• Extending the background, focusing on what the state of XAg is, what techniques are more prevalently used, and the benefits of using architecture-agnostic explainability methods; original PG explanations, how they are built, and scalability; and the relevance of intentionality and teleology in explanations.
• Presenting two use cases where they have been applied successfully and extending them with more results: a game environment (Overcooked) with RL and agents trained to imitate human behavior^25^ and a real-world driving scenario built on a human driving dataset with limited samples.^26^
• Providing new and extensive PG construction and design heuristics for generating the base PG in preparation for making an IPG*.* This extends previous heuristics in the literature^20^^,^^22^ that do not account for intentions.
• Summarizing the existing results on explainability based on desires and intentions introduced in the original work^25^ and extending the downstream uses of the revision pipeline introduced in that same work.
• Aggregating old and new metrics for evaluating IPG adequacy and performance, together with more explanations of how to interpret those metrics and their trade-offs, as well as justifying the need for intention metrics as opposed to using only goal-agnostic state criticality metrics used in the literature.
• Presenting the results of the two use cases and showcasing the usefulness of the revision pipeline for improving the agent behavior. We show examples in which these tools can be applied to justify and discover agent behavior and opportunities to control and improve it.
• Finally, we discuss our main contributions, possible future work, and known limitations of the approach, including the potential application to downstream tasks, such as collaboration and/or competition in multi-agent (MA) systems, human collaboration, and especially auditing of such systems.^20^^,^^27^^,^^28^

• Providing a workflow for creating and using an intentional PG (IPG) in new use cases, as described in Figure 1, also guiding the process of understanding IPGs*.*

## Background

The European Union imposes a right to explanations for automated decision-making systems that significantly affect users.^29^ Despite there being plenty of research encouraging the development of more transparent systems from even the knowledge-based systems community,^30^^,^^31^^,^^32^^,^^33^^,^^34^ the definition of what makes a system explainable is vague. There already exists a standard,^35^ and there have been attempts to match the regulation with its intent,^36^^,^^37^ with some emphasis on what makes an explainable system better or more trustworthy than one that is not and which are the properties of the umbrella-term explainability that foster these improvements.

One very prevalent issue is that the people generating explanations are often the system’s programmers, as opposed to the target explainees.^4^ The latter have little control of the sort of explanations that are provided, other than participating in user studies in which they compare contrastively which form of explanation (of a given, limited set) is preferable, as standards and papers recommend the introduction of human-in-the-loop to increase system trustworthiness.^38^

Instead, it is useful to take a step back to consider what insights from other disciplines tell us about good explainability.^4^^,^^39^

Firstly, it should be considered how to follow the four maxims for collaborative communication.^7^

(1) Manner: the message or explanans should be comprehensible and clear to the receiver, which, within the context of XAI, is often referred to as interpretability.^2^
(2) Quality: the message contains truthful information; in the context of XAI, reliability, or explanation verification.^8^^,^^9^^,^^10^
(3) Quantity: a message should be as long as necessary to be informative, a principle often embedded in explainable system design.
(4) Relation: the explanation should be relevant to the given context, avoiding endless searches for deeper causes.

These maxims bring clarity to the objective of XAI in general. Although the most prevalent motivation for designing explanation-generating methods is for the message to be interpretable to the receiver, there are obvious other properties that are sometimes taken into less consideration.

This has been a problem, as some user studies focus only on interpretability through qualitative analysis or user studies, which are, by nature, limited to a few examples and small cohorts of explainees. Sometimes, methods are picked based on interpretability—and thus generate more trust—as opposed to which method is actually telling the truth (i.e., quality or reliability). Reliability and truthfulness are irrespective of end explainees and can be evaluated in more quantitative ways too,^10^^,^^40^^,^^41^ and sometimes this obvious property has been ignored in favor of interpretability.^10^

While user studies are an important part of ensuring safe and explainable models, the belief that user studies are the sole grail for explainability evaluation has been recently challenged,^40^^,^^42^ with reasons including the possibility of other, more feasible metrics (or properties) to find good explanations but also the limited extrapolation that can be made of results when switching models, explanation methods, architectures, explainee background, or task domain.

Particularly concerning is the arrival of the age of large language models (LLMs) and the proposal to extract explanations from models that have been shown to be unreliable at best but are considered highly trustworthy by the general populace.

Altogether, this motivates a push for explainability that can do the following:

• It can run on *any* new model by being agnostic to its architecture or model.
• By virtue of being architecture-agnostic, its results can be compared between applications (i.e., domains, particular questions, and cohorts) and architectures seamlessly, making user studies on it more broadly applicable.^43^^,^^44^^,^^45^
• It can be quantitatively evaluated in terms of reliability.
• It takes into account insights from the social sciences on how to make useful explanations.
• It can modify its behavior based on the explainee’s preferences, i.e., responding only in terms that an explainee can understand and allowing the explainee to set and modify those terms.

### XAg taxonomies

On the topic of agent explainability, some surveys enumerate, categorize, and analyze the different existing methods and methodologies into several taxonomies.^11^^,^^21^^,^^46^^,^^47^^,^^48^^,^^49^ Each axis for grading an explainability technique shows a trade-off between the flexibility (i.e., how useful it is for a particular problem and if it can be applied to more or less agent architectures) and the complexity and usefulness (i.e., how much information the technique requires to function and what sort of information it can provide back) of the technique.

One way to categorize explainability methods is to distinguish between those that are intrinsic and those that are *post hoc*, based on the time of information extraction.^11^^,^^21^ Intrinsic methods build models that are inherently interpretable or self-explanatory during the design or training of the agent’s policy. Post hoc methods, on the other hand, focus on building the explanations by analyzing a policy that is already implemented or trained.

Related to the intrinsic/post hoc categorization, it is also possible to classify explainability methods into model specific and model agnostic.^11^^,^^21^ The former are tailored to a specific model or family of models, while the latter aim to be used for any agent policy. Most of the approaches found in the literature are model specific, either by having access to a full or approximate model of the agent or directly designing it^50^^,^^51^^,^^52^^,^^53^^,^^54^^,^^55^^,^^56^^,^^57^ or by possessing knowledge about specific important parts of the agent’s design, such as the reward function^58^ or the internal task decomposition.^59^^,^^60^ There are, however, methods, such as SHAP (Shapley additive explanations)^61^ or LIME (local interpretable model-agnostic explanations),^62^ that are model agnostic.

Another possible categorization concerns the scope of each explanation^11^^,^^21^: whether the method explains the entire behavioral model of the agent and therefore offers global explanations or instead offers local explanations in the sense that they target a specific decision. That is, global explanations help explain the model, while local explanations help explain a specific decision.^63^ Another aspect that can be considered when characterizing an explainability method is the part of the agent’s architecture that should be explained.^48^ Feature importance methods aim to quantify the influence of an agent’s input features (e.g., sensory information or percepts) on its decisions. Learning process methods bind the decisions to specific components of the design or training method that led to the policy, such as the reward function, the Markov decision process (MDP), or the datasets used. Meanwhile, policy-level methods aim to build a model of the agent’s long-term behavior.

For our work and given the initial premises that define its scope, we propose to focus on methodologies that are as follows:

• Post hoc, so that no assumptions need to be made about the design or training process.
• Model agnostic, to analyze opaque agents.
• Global and local, as we have two objectives: (1) producing a stable, comprehensive model of behavior^20^ and (2) allowing explanations of particular action decisions tied to long-term processes.
• Policy level, as we care not only about the reasons for a particular behavior but also about the relationship between the behavior and the environment.^48^

Explaining an agent’s behavior requires understanding both individual actions and their long-term purpose as trajectories rather than analyzing decisions in isolation. This often depends on an understanding of the environment in which the agent exists. In our work, we use PGs—a post hoc, model-agnostic, and policy-level explainability method^20^^,^^22^^,^^24^^,^^64^—to capture both local (action-level) and global (long-term) agent behavior.

### PGs

A PG is an explanation-generation method^20^ that models agent behavior and environmental dynamics by learning two key probability distributions: the agent policy, or *P*(*a*|*s*), which represents the likelihood of choosing action *a* in state *s*, and the environment’s response, or *P*(*s*′|*a*, *s*), which captures how the environment transitions to state *s*′ when action *a* is taken (often called a world model^62^^,^^63^^,^^64^^,^^65^^,^^66^^,^^67^ in the context of sequential decision-making [SDM]). These components share similarities with an MDP, often used in RL, notably, the Markovian assumption (i.e., the next state is independent of states and actions previous to the current state), and the probability distribution of the next state (*P*(*s*′|*a*, *s*)), but this is where the similarities end: there is no notion of reward or value functions in PG, nor is the action-taking mechanism treated as a problem to be solved but rather as a distribution estimated via observation. The PG is only these two distributions (as well as *P*(*s*), introduced in later works^25^).

In the paper initially presenting PGs,^20^ the objective was to produce explanations of robot behavior for humans, being mindful of the need for a human-robot common language to reduce the difficulty of understanding. To do this, an initial process of discretizing the state is performed. A discretizer (in this context) refers to a function that automatically transforms the observation space (i.e., what the PG is collecting as part of observing the states) into a discrete representation, originally as logic predicates (e.g., near the delivery area or south of the goal). The reason for this discretization is 2-fold.

On the one hand, it allows the explainee to set their preferences for what the response looks like, as was specified in the last requirement exposed in background. This disambiguates the interpretation of the elements in a PG: instead of requiring further hypothesizing of what the features of the input of the agent mean, a clear definition for each received predicate is presented (e.g., near the delivery area means that the robot is within two spaces of the delivery area in Manhattan distance). This property necessitates human feedback and should not be automated, as this removes the influence of an explainee in the explanations they receive.

On the other hand, it facilitates learning the two distributions (particularly *P*(*s*′|*a*, *s*)). These distributions could be learned via statistical methods (e.g., a deep learning model), but that merely shifts the explanation problem to the new model (which may even be unaligned to the original). Instead, by discretizing the state space, it becomes a tractable problem to estimate the distribution in a frequentist manner: computing the probabilities for *P*(*s*′|*a*, *s*) and *P*(*a*|*s*) between state regions as opposed to particular states. If the discretization were done only for this purpose, the process could be automated. In fact, it can be done using decision-tree approaches to distinguish between continuous states based on the differences in actions taken.^22^ Other approaches for producing predicates by automatically discretizing environmental state spaces or the agent perceptions include object segmentation techniques^68^ or knowledge or scene graph generation methods.^69^ Furthermore, should enough observations be available, this discretization can be made as expressive as the original state (e.g., an identity function).

However, the notion of the need for a discretizer (be it more or less expressive) for estimating the distributions brings forward the problem of scalability and applicability to complex domains, as well as the number of observations needed, given an agent and task, to build a reliable PG. As the (discretized) state space is much bigger than the action space, the number of observations for learning *P*(*s*′|*a*, *s*) sets the number of samples. Given the similarity to MDPs and RL techniques, there are some theoretical estimates^70^^,^^71^ that can guide the agent designer. However, there is strong evidence to argue that this problem can be likened to knowing the amount of data an ML model needs. It is too dependent on the qualities of the model and environment, so it is preferable to use methods and metrics instead to estimate *when* the amount of data is sufficient for the given purpose, as we will propose in the metrics section.

Once the probability distributions are computed, the PG is used to provide natural language answers (built from the predicates of the discretizer) to queries such as identifying conditions for actions (when do you do a?), explaining differences in expectation (why did you do a in state s?), and understanding situational behavior (what will you do when X is given?).^20^

However, the answer to these questions is inherently restricted to immediate results, as it does not provide answers to long-term action behavior, and it is agnostic to the agent’s goals, desires, or values. Later work used the properties of an automated discretizer to find state regions with consistent agent behavior (i.e., always performing the same action), naming them critical states,^22^ and for generating natural language answers to the same questions above.

Similar approaches to PGs that also use predicates have been applied to agents that follow a clear SDM process to achieve their goals. Some works^58^^,^^60^^,^^72^^,^^73^ advance on this approach, where agent behavior is modeled as a series of steps or plans. Unlike SDM-based methods, however, PGs do not assume any specific internal model for the agent or its decision-making process. This makes them more adaptable for scenarios where agents might have multiple goals or where their decision-making is not solely goal oriented. This flexibility is crucial for understanding agents whose behavior does not necessarily follow a straightforward path or cannot simply be assumed due to opacity.

Previous literature^23^^,^^24^ extends PGs to MA scenarios in which an agent trained with RL cooperates, either along with another RL agent or along with an agent trained to imitate a human player. An interesting consequence of the methodology is the creation of surrogate agents^24^: agents that enact policies automatically derived from the generated PG. These agents exhibit behavior comparable to that of the original trained agent, allowing this method to create policies that mimic the original policy while remaining transparent. This is a form of surrogate-agent modeling, such as those traditionally used for opaque ML models.^11^

Finally, with the introduction of IPGs,^25^ an extension is made to the algorithms of a PG that allows us to compute agent intentions. Intention is a key concept for telic explanations and is formalized consistently with previous literature on the topic,^74^^,^^75^ and we are aware of insights from the social sciences. Intentions are tied to a desire, expressed in terms of actions and predicates. For the same reason that a discretizer is not automated to give the explainee control over the explanations received, desires are not automated either: instead, prospective explainees collaborate with the system designer to determine which behaviors appear desirable, and explainees will understand whether they appear as part of answers to explainability questions. Intentions are the focus of this work, and in the following sections (particularly explainability based on desires and intentions), we provide further explanation of the algorithms and the formalization of desires, intentions, and how they can be used both for explainability and debugging an agent.

### Social sciences and intentionality

Many existing models explain agent behavior by identifying predicates relevant to individual action choices. However, this is not how humans typically seek explanations.^36^^,^^76^ Instead, people interpret behavior in terms of end goals, desires, and rewards—seeking to understand why an action contributes to an objective, what causes the objective to emerge, or how environmental affordances shape decisions about which objectives to pursue. Any such interpretation of behavior is what we refer to as a telic explanation.

These meaningful explanations require clear notions of an agent’s objectives, often necessitating theory-of-mind-inspired algorithms.^77^^,^^78^ Additionally, trajectory-based reasoning enhances predictability by considering sequences of actions rather than isolated decisions. Trajectories can be defined as sequences of state-action pairs that characterize an agent’s (*A*) behavior. For example, the sequence *A* boils water, then *A* cooks the pasta, then *A* adds sauce to produce pasta carbonara is more informative than viewing each action in isolation, as it reveals a pattern that likely leads to an overarching goal—putting in something to cook is a very likely action after putting water to boil, and that eventually leads to having prepared pasta carbonara.

In the control-justify-improve framework,^11^^,^^19^ behavior predictability enhances explanation relevance. A key approach to improving predictability is by analyzing intentionality.^30^^,^^78^^,^^79^^,^^80^ Intentions are mental states distinct from other states such as beliefs, desires, knowledge, or emotions. The content of an intention is a state of affairs that will be the aim of the agent and to which it commits.^81^ However, attributing intentions to opaque agents entails significant risk, thus a burden of attribution, and demands careful validation. While this attribution may not be entirely accurate from a formal perspective,^5^ it is practical and beneficial to do so—since humans constantly engage in this attribution process to explain events, this burden can often be overlooked. The topic of intentionality and how to deal with intentions and their attribution from a practical point of view will be developed in detail throughout explainability based on desires and intentions section, and to evaluate how these theoretical insights can be operationalized, we then apply our method to concrete use cases in the results section.

In the literature on folk-conceptual theory of behavior explanation, there is a clear distinction between two different ways humans explain behavior: intentional and unintentional.^76^ The difference lies in whether the actions to be explained appear to be done with a purpose or not. Actions that appear purposeless or unintentional can be explained with mechanical causal factors.^69^ Most previous work on PGs^20^^,^^22^^,^^24^ focuses on answering queries of this sort: listing beliefs of the agent and state, which would fall in the kind of explanations usually provided for unintentional behavior. The same applies to most feature attribution techniques such as SHAP^61^ and LIME.^62^

Within those that have a purpose, intentional explanations have been further classified into three categories.^39^

• Reason explanations (REs), are concerned with the causality of an action being taken as assigned to “what the intention is, and how an action favors it” and are by far the most common kind (3 in 4 cases).^39^^,^^74^^,^^75^ In a context of BDI,^82^ it can be likened to answering in terms of “what the current goal (or desire) is, and what beliefs tie the action to the success of the goal.” In addition, this type of explanation often includes additional reasons, such as avoiding alternative outcomes or beliefs about the context. An example could be the following: “Why are you studying?” “Because I have an exam tomorrow, and I want to pass the subject.”
• Causal history of reasons (CHR) explanations, which are concerned with explaining the precursor factors to the reasons an action is chosen (including intentions). In other words, the cause of a goal or desire that is being pursued. In the context of RL, this is intrinsically—but not exclusively—tied to the choice of reward function. As an alternative example, in BDI, responses would be tied to the designer’s choice of desires and values. An example could be the following: “Why are you studying for tomorrow’s exam?” “Because I need to get my degree if I want to get a high-paying job.”
• Enabling factor (EF) explanations, which concern themselves with explaining why an action that is apparently desirable was successful. A question is generally understood as an EF when the motivation appears to be clear but the means are unknown. An example could be the following: “Why did you get a 10 in that difficult exam?” “I studied for weeks before taking it.”

## Use cases

As previously stated, a key objective of our proposed method is its applicability to any opaque agent, regardless of its underlying architecture or training process. To evaluate this, we selected two distinct use cases. Initially, our test bed was Overcooked-AI,^83^ due to its well-defined performance and achievement goals, which enable controlled assessment. Second, we applied our method to nuScenes,^84^ a dataset for autonomous driving (AD) research, to prove the method’s scalability and effectiveness in a complex, real-world domain. These case studies serve to validate the framework in both controlled (game) and real-world (driving) environments.

### Overcooked-AI

Overcooked-AI is an MA RL environment where two agents must collaborate to prepare and deliver as many dishes as possible within a fixed time limit. This cooperative setting gives rise to emergent behaviors not observed in single-agent scenarios, making it particularly relevant for studying explainability.

The Overcooked-AI environment provides multiple layouts and configurations, each encouraging different optimal strategies and behaviors. By generating PGs for agents trained in distinct layouts and analyzing them through static and intention-based metrics, we can derive meaningful insights into their decision-making processes.

**Figure 2:**
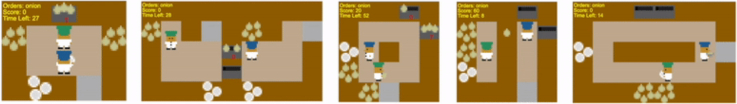
Overcooked visualization of the analyzed layouts From left to right: simple, unident_s, random_1, random_0, and random_3.

This environment is versatile and can target several tasks, layout arrangements, and affordances. Five of the most used layouts (i.e., the layouts introduced in the paper where the authors present the Overcooked-AI environment^83^) are considered for displaying the PG usage and our proposed metrics. All layouts consist only of the delivery of onion soup. An agent completes this task by adding three onions to a pot, which produces soup after a few time steps. An agent can collect the soup with a dish and deliver it in a specific “service” tile. Figure 2 is a graphic visualization of these environments.

Each agent in the environment occupies a tile in a 2D grid-like map and faces a specific direction. Two agents cannot occupy the same tile. Agents have six possible actions:

• Moving in one of the four directions (therefore four possible moves) changes the direction they face and, if the tile in that direction is unoccupied, moves them to that position. The confrontation is resolved stochastically if two agents attempt to move to the same position.
• Interacting with the element in front. This action encompasses several possible actions depending on the context: picking up an item, placing it in the agent’s hands or on a counter, putting an onion into a pot, using a dish to scoop soup from a cooked pot, or delivering the soup to the service area.
• Staying idle, which does nothing and lets the time step pass.

Each layout requires unique strategies, often benefiting from agent collaboration.

• “simple” is a cramped room where agent positioning may hinder the other agent. It has a single pot, unlike all other layouts.
• “random_1” and “random_3” require agents’ coordination to avoid getting stuck in thin corridors. With the longer table in random_3, agents would benefit from passing onions over the counter.
• “unident_s” has each agent in different isolated regions, and each side has a different distance between affordances. Agents would benefit from specializing (i.e., left agent for servicing and right agent for cooking).
• “random_0” similarly has each agent in different isolated regions, but each affordance is different, forcing collaboration. The agent on the left needs to pass onions and dishes over the counter to the agent on the right.

Agents receive positive rewards for successful soup deliveries, but any intermediate action—e.g., picking onions or carrying dishes—yields no explicit reward. These rewards provide guidance for shaping the agents’ learned behavior, but ultimately, rationality and adherence to any optimal strategy will vary among agents depending on their training process—e.g., algorithm, hyperparameters, and training time. Within the scope of this paper, we intend to analyze and characterize agents that are not optimal or perfectly rational rather than just validating their rationality. Therefore, rather than selecting and training specific agent types or optimizing for performance, we chose an existing set of heterogeneous pairs of pre-trained agents with distinct behavioral patterns and performance levels.

• Agents trained from scratch with proximal policy Optimization (PPO), collaborating in the same training environments.
• An agent trained from human trajectories exclusively and a PPO agent trained to collaborate with it.
• A random baseline, based on a combination of a PPO agent and an agent that takes random actions.
• More details about these agents, how they were trained, and the rationale behind the selection can be found in the results section.

### (Human) AD

The nuScenes dataset^84^ contains scenes of human drivers navigating real-world urban environments in Boston and Singapore. The dataset predominantly captures ordinary maneuvers (e.g., lane changes) and activities in dense traffic situations, such as navigating intersections and pedestrian crossings. In addition to these common scenarios, nuScenes includes a subset of rare and intricate situations, including interactions with emergency vehicles, animals, construction zones, and hazardous events such as pedestrians jaywalking.

To encourage diversity in the dataset, data collection was conducted at different times of the day and under different weather conditions. This variety of scenarios and realistic interactions makes nuScenes a particularly compelling use case for explainability research in real-world AD applications and in modeling human driving behavior.

Each scene has an approximate duration of 20 s and is recorded using a comprehensive array of automotive sensors, synchronized at a frequency of 2 Hz, resulting in a total of 40 frames per scene. Each frame contains comprehensive information about the vehicle’s state, including its position, velocity, and acceleration, as well as internal vehicle data such as turn signals and steering angle. Additionally, vehicle state information is augmented with annotations and state information of surrounding road participants, including other vehicles, pedestrians, cyclists, and road objects such as bicycle racks and traffic cones.

The dataset comes with a static map of each driving location, covering the topology and geometry of road features such as parking areas, pavements, pedestrian crossings, road signs, and intersections.

In this use case, the opaque agent is the human driver, and the objective is to extract explanations of the driver’s behavior from observational data collected during driving scenes. Notably, no agent training is performed; the study considers only the recorded behavior in the dataset.

## Methods

PGs are not off-the-shelf solutions; they require careful design, including defining a structured state representation and verifying its correctness. In exchange, the resulting artifact is highly flexible, providing explainee-customized local and global explanations of policy behavior,^20^ can work as transparent surrogate models,^23^^,^^24^ and allows iteration on responses to increase understandability and justify PG-provided answers.^25^ An overview of PGs is provided in the background.

Our approach to IPG construction involves two key human-dependent steps: first, creating a descriptive code for states to ensure interpretability and expressiveness (i.e., a discretizer) and second, formulating hypotheses about the agent’s desirable behavior within the framework. This methodology balances interpretability and reliability, iteratively refining representations to improve the clarity and accuracy of agent behavior explanations. Figure 1 provides a depiction of the proposed workflow.

First, a representative sample of observations of the target agent acting in the environment must be collected. We recommend storing all available information before its discretization, as the pipeline may encourage the designer to change the discretizer: the explainee’s questions and perspective may evolve. In the case that storing everything is not viable (e.g., original states or trajectories are too large to store), trajectories should be stored as expressively as possible to increase flexibility when designing discretizers. By “as expressively as possible,” we mean storing as many of the original observations as possible. For example, in the case of Overcooked, it is recommended to store the relative position of the other agent if it cannot be derived from the rest of the predicates. Even if the first planned discretizer will not use it, it may later appear to be a useful predicate, and obtaining it would require discarding the old trajectories to take new ones. The decision is a trade-off between storage space and model expressivity. In PG construction and design heuristics, more information is provided on how to design discretizers.

Once observation data are obtained and a discretizer is selected, a base, non-IPG is created by computing and storing probability distributions: *P*(*s*) and *P*(*s*′, *a*|*s*), that is, the probability distribution of being in a discretized state *s* and the transition probabilities when in that state—what the agent does, *a*, and what happens to the state, *s*′. Note that it is trivial to compute *P*(*a*|*s*) and *P*(*s*′|*a*, *s*) from *P*(*s*′, *a*|*s*) via marginalization and the Bayes rule.

The PG can already be validated in several aspects using static metrics. These allow the practitioner to determine whether the discretizer is adequate, given the amount of data available to build it. There are separate metrics to evaluate how informative *P*(*a*|*s*) and *P*(*s*′|*a*, *s*) are, how well the PG predicts behavior (of the agent and the environment) in unseen data, and how well a surrogate agent made from the PG can perform if it substitutes the agent. Each of these can be used to iterate on the discretizer design, as they provide measures of whether the discretizer is too simple or too complex, models useless predicates, or lacks key predicates.

To convert the PG into an IPG, desires are introduced, which can be converted to numeric intentions for each desire and state. This allows us to run telic explanation algorithms. In addition, numeric intentions can be validated with intention metrics, which allow us to measure the existence or absence of the desires. Furthermore, we argue for the necessity of human-aware desires, as opposed to automating everything, e.g., via critical states^22^ (see are static metrics not enough?: the traffic light thought experiment).

Finally, the revision pipeline is presented as a task that serves as a real-time explainability chart and showcases how it can be used to improve the IPG (e.g., by adding new desires) and debug/improve an agent or MA system (e.g., by finding regions in which the agent acts irrationally).

### PG construction and design heuristics

Building a PG involves observing an agent’s behavior and discretizing it into a finite state space. The function performing this task is called a discretizer. While it can be obtained automatically,^22^ for the reasons of customizability and explainee adaptation described in the background, we suggest against doing so. While many formalisms can represent the discretized states, we recommend the following properties.

• The state space is a metric space where we define a distance function that computes the similarity between states. Generally, this is done with a simple count of different predicates,^20^ but more sophisticated approaches that account for predicate semantics could provide better explanations (as of now, this only applies to the original PG question answering^20^ and not to IPGs).
• The resulting state space is sufficiently general (i.e., descriptive but reduced in number of states) that the agent can map states from new observations to existing, already observed, discretized states. This can be checked with static metrics.
• The resulting state representation should be interpretable by human explainees or by relevant downstream tasks. More specifically, the non-discretized state’s properties should be interpretable based on its discretized version’s internal representation (i.e., the discretization should be understandable). This understanding can be incomplete so long as it allows for the justification or interpretation of agent behavior.
• The resulting state representation allows for formally representing desires*.* Parallel to designing the PG, it is recommended to hypothesize about the desired behavior. The IPG introduces the possibility of desires in terms of discretized states: if a desire is considered, then the discretizer should allow it to be expressed. For example, if a human wants to hypothesize a desire to pick up an object when it is close by, the discretizer will be required to encode the property of an object being close by.

The rationale for these heuristics can be understood from the trade-off between interpretability and reliability.

On the one hand, the first two properties are intended to increase reliability. The probability distribution represents the real world only if the observations are sufficiently frequent in the graph. In addition, by introducing a notion of distance, one can treat the state space as a metric space and use similarities between states to compensate for the lack of observations at the cost of some reliability. The issue of the amount of observations needed to obtain a reliable representation has been tackled in some work that estimates similar components (see proposition 1 in this work, Zeng et al.,^71^ or Topin and Veloso^70^). As theoretical upper bounds, these formulas may overestimate the number of samples required to learn a reliable representation, so a practitioner may want instead to use static metrics to evaluate how reliable their representation is (akin to how a ML practitioner evaluates if they have enough data based on validation or test accuracy).

On the other hand, the representation of the internal states will be part of the code shared between the explainee and the model. If such code is not shared, the result will be challenging to interpret. This, in turn, allows for explanations that conform to what the explainee can understand.

Both necessities go in opposite directions: having a small state space hinders the expressivity demanded by an extensive code of communication between the explainee and the model, thus hindering interpretability. Similarly, a thorough state description implies a larger state space, in which the specificity of each state results in a lower probability of reaching it during observation. In turn, this lowers the reliability of the probabilities conditioned on being in such a state. This is a significant problem when working with real-world problems with scarce available data, as it requires finding a state representation that is sufficiently detailed to produce explanations. This complexity also explodes when considering that states requiring explanations (e.g., those that surprise or confuse an explainee) are often less frequently visited, thus increasing data-gathering requirements.

Handling the trade-off between interpretability and reliability depends on the task at hand, thus requiring metrics to evaluate which side is favored by a specific discretizer or representation and to choose accordingly. For example, in high-stakes environments, the truthfulness of answers is imperative. It is preferable to know that the algorithm cannot truthfully explain some situations than to receive dubious explanations (e.g., explanations that underestimate the probability of a transition). Instead, in a low-stakes environment, it may be preferable to receive many explanations, even if some are misleading.

Finally, although anyone, including non-experts, can propose discretizers, their usefulness depends partly on the state-space description. Experts in the field are more likely to correctly assess which environmental parameters are more relevant to the agent’s behavior and thus be more efficient in their search for the optimal discretizer. Still, the metrics proposed and the pipeline described in Figure 1 allow non-experts to bridge the gap through more iterations of the process.

Following previous work,^23^^,^^85^ we pick a simple discretizer and distance that are directly matched with our representation. We describe each state using problem-specific propositional logic predicates, discretizing real states by evaluating the truth values of each predicate and assigning the corresponding discretized state.

We take the number of different predicates between two representations with no weighting for distance. We note that more sophisticated representations exist, such as employing decision trees,^22^ using clustering on state CLIP (contrastive language-image pre-training) embeddings or even scene graphs.

For the problems tackled in this article, a straightforward approach successfully provided explanations out of the box for both use cases, reusing discretizers for Overcooked^23^^,^^25^^,^^85^ and AD.^86^ Each of these is detailed in its respective discretizers and static metrics sections.

### Explainability based on desires and intentions

Most explainability algorithms in the literature focus on establishing some causal relationship, correlation, or relevance between some input variable and the model’s output.^61^^,^^62^^,^^87^ However, when asking a human why they put a pot on the hob, it is doubtful that they will reply, “The pot was full of water, and the hob was not being used.”

A correlation may exist between a pot full of water and the cook placing it on top of the hob, as cooks often fill the pot with water when they plan to boil it. However, the motivator of such behavior is not the availability of the pot and the hob but the intention of the task. Since humans can *consciously* set goals, explanations of their intent are often teleological, focusing on the purpose behind the behavior (e.g., because I want to cook some pasta). In many cases, these teleological explanations encompass the realms of morals, ethics, and politics,^5^^,^^88^ but the actual intention acts as the main predictor of the existence of abstract mental states such as holding a particular value or moral norm^89^ (e.g., self-preservation). In our example, an explanation a human cook would give to someone who does not know how to cook would more likely be, “To make pasta carbonara, I need to cook the pasta, which requires boiling water.”

Although further explanations may involve state variables such as the state of the pot or the hob, the natural communicative act cannot constrain itself to that level alone.^55^

When analyzing a (reasonably well-performing) agent’s behavior in a domain, humans tend to anthropomorphize.^16^^,^^17^^,^^18^ So long as the agent’s actions are not entirely random and there is a way to establish logical inferences from them from a teleological perspective,^5^^,^^90^ humans attribute intentionality to the agent (e.g., it has grabbed the onion because it intends to put it in the pot later). This is especially the case for most toy environments (e.g., games) for which the human observer has some knowledge of how to solve and thus expects certain behaviors from their virtual homologs. It extends to experts observing agents’ behavior in their domains.^91^^,^^92^

With limited observations, these attributions may be anecdotal unless systematically verified. In this section, we present a way to leverage this cognitive bias to enable agent explainability to answer the what, why, and how questions in a manner not dissimilar to how a human would. We introduce agent desires—which can be modeled in various ways—and agent intentions—desires the agent is expected to pursue and accomplish (soon) as allowed by the environment.^81^ In addition, we introduce to this pipeline a hyperparameter that directly lets the human control the interpretability-reliability trade-off: the commitment threshold.

#### Desires

In this work, desires are introduced as hypotheses over expected behavior: the work of anthropomorphism by a human observer who has some rudimentary or expert knowledge of the task the agent is solving. A desire may or may not manifest in the agent’s behavior, necessitating verification. If a desire manifests in an agent’s behavior, it is often due to design factors such as system rules, reward function design, or statistical biases in the training data.

Pragmatically, to define a desire, we must determine when it is fulfilled. We distinguish between several cases, such as reaching or staying (achievement and maintenance goals, respectively, as shown by van Riemsdijk et al.^93^) in states where some qualities hold (e.g., in Cartpole, to stay in a state where the rod is upright), to execute an action in such states (e.g., in Overcooked, to interact with the service zone with soup in my hand), or performing a particular transition between world states (e.g., in racing, crossing the finish line). These also extend to their negative forms, such as “not” staying in some states.

We concentrate on the second type: action focused. With strategic discretization, many desires can be framed in this way,^24^ and extending the framework to other types of desires is possible. Action desires can thus be defined as a tuple ⟨*S*_*d*_, *A*_*d*_⟩ containing a discrete state region (*S*_*d*_ = {*s* ∈ *S*|*s*⊧*d*}, where *s*⊧*d* means that the state satisfies the desire’s condition) and the set of actions *A*_*d*_ that would be desirable in such a state region. As the explainees themselves provide this characterization, they are expected to understand it when it becomes the finality of explaining behavior.

**Figure 3:**
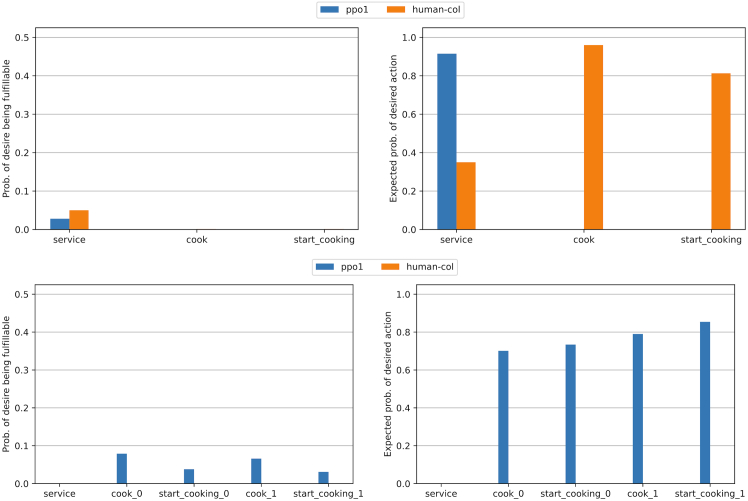
Desire metrics in Overcooked-AI Desire metrics for two types of agents (human-collaborating agent and PPO agent 1) in simple (top) and unident_s (bottom) layouts and the same discretizer (1), all described in agents used: Overcooked-AI and discretizers and static metrics: Overcooked-AI. The probability of a desire being fulfillable (left) is very low for all cases. Higher probabilities of desires being fulfillable are indicative of higher performance, subject to the desire being actually fulfilled (right).

Calculating relevant information over these desires is trivial under the probabilistic description of a PG. “How likely are you to find yourself in a state where you can fulfill your desire by performing a desirable action?” can be computed as the desire state region probability $P\left(s\in S_{d}\right)=\sum_{s\in S_{d}}P\left(s\right)$. “How likely are you to perform a desirable action when you are in the state region?” can also be computed as $P\left(a\in A_{d}|s\in S_{d}\right)=\sum_{a\in A_{d}}\sum_{s\in S_{d}}P\left(a|s\right)*P\left(s\right)/P\left(s\in S_{d}\right)$. These metrics can be found for some of the experimental environments in Figure 3 (the description of each desire can be found at the end of this section), and they serve as a first verification of the desires. PPO agent 1 unident_s never fulfills the service desire but is quite frequently fulfilling the rest. Note how the human-collaborating agent is never in a state in which it can fulfill any hypothesized desire in unident_s, meaning its behavior is inexplicable. Each graph represents an agent’s desires, evaluating the same desire for each agent. Except for the first one (human-collaborating agent), at least one of their desires is shown not to exist, as the desirable action is never performed in the state region, illustrated by the lack of expected action probabilities.

The addition of desire and desire metrics by themselves is not a panacea for the problem. Most states in a problem do not manifest the specific conditions for immediately fulfilling a desire, as *P*(*s* ∈ *S*_*d*_) is expected to be low in most cases. The reliability of the obtained metrics is directly measurable by *P*(*a* ∈ *A*_*d*_|*s* ∈ *S*_*d*_) (i.e., explanations expressing that the cause of a certain behavior is that the agent is willing to fulfill the desire can be wrong if an action of the desire is not performed).

That being the case, given that only states in a desirable region can be interpreted—and those states often account for a tiny slice of time—the agent’s behavior cannot be safely interpreted most of the time. For this purpose, intentions are introduced as an extension of this framework.

Using the case of Overcooked as an example, the following desires are guessed and tested, formalized using propositional logic.

(1) The agent desires to service soup: the state region is all states where the agent can deliver soup (that is, all states where the agent has soup and the service zone is in the interact position) and the action to be performed is to interact.
(2) The agent desires to cook: the state region is all states where the agent can add an onion to a pot with already one onion in it (i.e., having an onion, the pot being in the preparing state, and the pot being in the interact position) and the action to be performed is to interact.
(3) The agent desires to start cooking: analogous to the desire to cook, but the state region requires the pot to be empty instead of preparing*.*

When proposing these desires in the first iteration, the intention was to seek high-granularity tasks to verify the explainability of the system on a small subset of desires. More desires could be formulated, such as the desire to grab an onion when the pot is empty or preparing, but these were enough to achieve good interpretability metrics.

#### Intentions

To answer why questions, we leverage a PG’s transitional information. An agent’s intention to fulfill a desire exists if it can be fulfilled (given by world dynamics and its understanding) and the agent commits to doing so.^81^ Our empirical observations of the agent’s behavior capture both requirements.

Loosely defined, intentions of fulfilling a desire *I*_*d*_(*s*) can be measured by considering the probability that the agent will attain the desire from a given state. Informally, it is the sum of probabilities of all possible paths starting in one state that arrive at any state where the agent can fulfill the desire and is fulfilled.

Formally, let P(s,d) be the (potentially infinite) set of paths starting from *s* and arriving at any *s*′ ∈ *S*_*d*_ (not counting paths that fulfill the desire midway through). The intention of such a desire can be thus computed as $I_{d}\left(s\right)=\sum_{a\in A_{d}}\sum_{p\in P\left(s,d\right)}P\left(a|last\_state\left(p\right)\right)*P\left(p\right)$, where *P*(*p*) is the probability of traversing path *p* as computed by the PG: $P\left(p\right)=\Pi_{s^{\prime },a,s_{t}\in p}P\left(s^{\prime },a|s\right)$. One could consider the metrics used to describe desires to be myopic intentions restricted to paths of 1-action length.

Given the potentially infinitely looping paths, the computation is done backwards, starting from *S*_*d*_ and recursively propagating intention updates to the parent states. A stopping criterion *ϵ* is introduced to stop the propagation of intentions below a certain probability. A complete description of the algorithm can be found in Algorithms 1 and 2.

Algorithm 1 **Register a desire into a PG and propagate intentions**

**Require:** *d*, *PG*

 **for** *s* ∈ *PG*, **do**

 *I*_*d*_(*s*) ← 0

 
**end for**

 **for** *s* ∈ *S*_*d*_, **do**

 
$increment\leftarrow \sum_{a\in A_{d}}P\left(a|s\right)$

 propagate_intention(*s*,*d*,*PG*,*increment*)

 
**end for**

Algorithm 2 **Propagate intentions to node s**

Propagation of desires is stopped from crossing through the transitions that would fulfill them, as not doing so would compute the “expected number of times a desire will be fulfilled” instead (which can be above 1).

**Procedure** Propagate_intention(*s*, *d*, *PG*, *increment*)

 *I*_*d*_(*s*) ← *I*_*d*_(*s*) + *increment*

 **for** *p* ∈ {*p* ∈ *PG*|*P*(*S*′ = *s*|*S* = *p*) ≠ 0}, **do** ⊳ All parents of *s*

 **if** *p*∉*S*_*d*_ **then** ⊳ P cannot fulfill the desire, all transitions are valid

 *propagable*_*intention* ← *P*(*S*′ = *s*|*S* = *p*)∗*increment*

 **else** ⊳ P could fulfill the desire by doing *a* ∈ *A*_*d*_, ignore those actions

 *propagable*_*intention* ← *P*(*S*′ = *s*, *A*∉*A*_*d*_|*S* = *p*)∗*increment*

 
**end if**

 **if** *propagable*_*intention* ≥ *ϵ* **then** ⊳ stop criterion, usually 1*e* − 4

 propagate_intention(*p*,*d*,*PG*,*propagable*_*intention*)

 
**end if**

 
**end for**

 
**end procedure**

The algorithmic cost of this operation is the most significant in the IPG construction pipeline, aside from taking observations of the agent. Its theoretical cost is difficult to analyze, as it depends strongly on the graph’s characteristics. Mainly, it depends on the highest probability of a cyclic path: the closer to 1 it is, the more times intention will be propagated through it until it falls below the *ϵ* convergence criterion. We suggest starting with a relatively high *ϵ* (e.g., 1*e* − 3) that introduces some error in computation for exploration and lowering it according to computational resource availability for experimentation. Computational efficiency matters are further discussed in limitations. It is important to note, however, that the algorithm only needs to run once unless changes to the discretizer are made. Adding a new desire does not require recomputing other desires, and removing intentions scales linearly with the number of nodes in the graph.

Introducing *I*_*d*_(*s*) as a tool allows the explainee to ask for complex queries. For example, one could ask, “What do you intend to do in state *s*?”, to which the agent could reply with all desires with an *I*_*d*_(*s*) over a certain threshold. Another question could be, “Why did you take action *a* at state *s*?”, to which the algorithm would reply, “I have the desire *d*, which I can bring about from the state *s*, and by performing action *a*, either I am closer to achieving it, or there is a chance I will increase my odds of doing so.”

This aligns with the previously introduced Gricean maxims. Quantity-wise, this communication summarizes a (potentially enormous) set of explicit beliefs (i.e., the transitions between states and the policy). Relevancy-wise, it answers the question’s object directly (*a* and *s*) with the immediate causes (*d* and *I*_*d*_) and, as we discuss in the following section (explanation algorithms), is responsive to further needs by inquiring about the causes of *I*_*d*_ should they be needed. Manner-wise, the response is in a format that humans are good at interpreting (intentional explanations), and *d* is assured to be understandable by the human (as they proposed it): *I*_*d*_ is the probability that some desire will be brought about given a state. Furthermore, should the provenance of *I*_*d*_ not be understood, we allow further questions to clarify, as done with relevance. Quality-wise, the definition of intention is such that future behavior is conformant to the explanation in a manner proportional to the value of *I*_*d*_ (i.e., explanations are more likely true with *I*_*d*_).

This last point implies that the lower the intention value, the more uncertain its fulfillment becomes. Moreover, the continuous nature of intentions entails that an explainee may convince themselves of wrong information by vastly overestimating a probability. Tim Miller et al.^4^ point out that probabilities in explanations can be confusing for humans. Also, Zach Burns et al.^94^ show that this difficulty is aggravated by well-documented individual differences in numerical and probabilistic reasoning capabilities. To mitigate this, we propose restricting intention attribution to those above a parameter we call the commitment threshold, 0 < *C* ≤ 1, which specifies at which minimum probability the explainee is willing to believe the agent will try to fulfill a desire. Any *I*_*d*_(*s*) < *C* is to be disregarded, whereas for any state *s* such that *I*_*d*_(*s*) ≥ *C*, the agent can be said to have (at least some) intention to fulfill *d*, and we can say that *s* is attributed to the intention *I*_*d*_. Note how, importantly, any state can have a number of intentions attributed simultaneously, as they are analyzed independently.

This commitment threshold parameter is directly related to the reliability-interpretability trade-off. When the parameter *C* takes on higher values, it boosts the likelihood that any state to which intention is attributed will fulfill the desire. On the other hand, when *C* is lower, more states are attributed with intentions, which makes a more significant part of the behavior interpretable. However, some intentions may go unfulfilled, leading to less reliable explanations.

We measure and control this trade-off by extending the desire metrics into “intention” metrics (dependent on *C*): the probability of intention attribution and the expected intention. These two metrics, which are approximations of interpretability and reliability, respectively, can be computed for each desire and the PG overall.

#### Explanation algorithms

To use the computed intentions effectively, we must identify key explainability questions and answer them in a way that conforms to what has been exposed in the background (in particular, in the social sciences and intentionality section). In this section, we focus on producing intentional explanations using the computed intentions and compare them with unintentional explanations produced by existing methods.

Consider, for example, a state *s* in the simple layout, in which an agent is in the bottom row, center, holding a dish, while the pot is finished cooking. The agent’s next action, *a*, is going to be moving up (*↑*). As a human explainee, we believe, based on the agent’s previous behavior, that it plans to interact with the pot to obtain soup, then deliver it to a service zone. We run several explainability algorithms that, as stated, fall within the unintentional explanations kind.

The SHAP algorithm^61^ provides the following output:

Given that the agent observed *S*, the features that had more relevance to choosing action *↑* are *holding a dish* (+1.29)*—pos*_*y*_(pot) = *pox*_*y*_(agent) (+1.17)*—pot cooktime* = 0 (+0.66)*—pot is full* (+0.38)*—pot is ready to serve* (+0.35)*—0 onions in pot* (−0.32), …

Similarly, LIME^62^ responds in similar terms:

Given that the agent observed *S*, the features that had more relevance to choosing action *↑* are *holding a dish* (+0.18), *onions in pot* ≤ 0 (−0.16), *pos*_*y*_(*pot*) = *pox*_*y*_(*agent*) (+0.14), *pot is full* (+0.07), …

While the explainee may make sense of these factors after some deliberation (e.g., holding a dish and the pot being full/ready to serve, discarding the relevance of the pot having no onions, etc.), to understand the mechanical causes for the action in a general sense, the task still requires substantial speculation when interpreting the answer and predicting future agent behavior from it.

This extends to other techniques such as layer relevance propagation,^95^ which would similarly report positive relevance on features such as “holding a dish,” “pot being finished,” and the current [*x*,*y*] position of the agent. The original PG^20^ could ask three questions: “When do you go up?”, reporting an extensive list of situations in which the agent usually goes up; “why not left (←) in *S*?”, reporting that it would do so were the pot not finished or the agent not holding a dish; and “What will you do when the pot is not finished?”, most likely not returning an answer per design, as there are too many possible actions within that scope.

None of these explainability systems reports the *function* of action *↑*, which is related to the task’s objective (delivering soup). Other types of questions (and, therefore, answers) must be provided for the intentional kind, such as when why means why for. An example of these would be the aforementioned example, “I boil water because I want to make spaghetti.” In this direction, the IPG explainability desiderata provides the following:

(1) “What do you intend in *S*?” Deliver soup.
(2) “How will you do it from *S*? Why do you believe it is possible?” (A detailed plan of action and how the world is expected to change, culminating in delivering soup.)
(3) “Why do you go up?” Because it helps me deliver soup.

The design focuses on identifying the questions that require answers, guided by two key principles of maxims^7^: the information provided should be minimal (in line with the maxim of quantity) while ensuring that sufficient question types and methods are available to pursue additional information if required (maxims of relevance and manner).

The three questions in our desiderata can also be related to the intention explanations from social sciences and intentionality.^39^ Within the context of RE, explanations for actions are related to “what the intention is, and how an action favors it.” The former is the answer to our first question (what), while the latter is tied to the second (why) and third (how) questions, with the third providing a much more extensive response. Regarding CHR explanations, these are concerns with the reason behind the desirability of a state; we argue that, as the explainee provides the desires based on some understanding of the agent’s functions, this question is already solved in the mind of the explainee. Finally, EF explanations could possibly be tied to the affordances and beliefs that bring the agent to manifest intention toward a desire, but this falls outside the scope of this contribution.

Now that the target questions have been defined, we propose some algorithms for generating responses. These algorithms are examples that are left in pseudo-natural language. Their purpose is to show that the information to construct a natural language explanation is available, but which particular presentation is best for each possible downstream task (e.g., presenting it to a non-technical explainee, using it to debug an agent, or passing the information to another agent for coordination) remains as future work.

The first question is the easiest one to solve: given a state *s*, returning any attributed intentions *I*_*d*_(*s*) ≥ *C* (Table 1). However, this needs a more satisfactory explanation. For an intention to exist, the agent needs to have the desire and believe that it can be fulfilled. Suggesting the former may not elucidate the latter, and as is apparent by the frequency of REs, it is a prevalent necessity. As an example, consider the Cartpole environment (https://gymnasium.farama.org/environments/classic_control/cart_pole/): if an agent returns that it intends to straighten the pole up in a state where it is falling left, we expect an answer such as the following: “My goal is to keep the pole upright. Currently, the pole is upright but leaning to the left, and I am not on the left edge, so I move to the left. This results in a situation where the pole is no longer leaning left, thus achieving my goal.”

**Table 1:** Answers to what and why questions in state 84 of a human-collaborating agent in the simple environment using PG-discretizer 1

What?	desire_to_service (0.82)
Why (Interact)?	I want to do Interact for the purpose of furthering desire_to_service as it has a 0.99 probability of an expected increase of 0.01.

To get an answer such as this, the second question reasons how the agent believes the goal will be achieved (Table 2). Algorithms 3 and 4 return increasingly in-depth answers to the query. Intuitively, the former returns the most optimal path to fulfilling an intention by picking the action and successor state (where a successor holds {*s*′ ∈ *PG*|*P*(*S*′ = *s*′, *a* = *a*|*S* = *s*) ≠ 0}) to the current considered state, such that the successor has the highest increment in *I*_*d*_; this is repeated until *d* is fulfilled. As the intention in a state is a weighted average of the intentions of its successors, it is always the case that either at least one successor has a larger or equal intention or the current state can directly fulfill the desire.

**Table 2:** Answer (deterministically) to the question of “how to deliver soup” from state 84 of a human-collaborating agent in the simple environment using PG-discretizer 1

Interact (0.82)	Right (0.89)	Down (1.0)	Interact (fulfilled)
HELD_PLAYER (SOUP) POT_STATE (POT0; ¬STARTED)	ACTION2NEAREST (ONION;INTERACT) ACTION2NEAREST (POT_0_; ←) ACTION2NEAREST (SERVICE; *↓*) ACTION2NEAREST (SOUP; *↓*)	ACTION2NEAREST (ONION; *↑*) ACTION2NEAREST (SERVICE;INTERACT) ACTION2NEAREST (SOUP; →) POT_STATE (POT_0_;PREPARING)	–
HELD_PLAYER (DISH) POT_STATE (POT_0_;FINISHED)	ACTION2NEAREST (ONION;RIGHT) ACTION2NEAREST (POT_0_;INTERACT) ACTION2NEAREST (SERVICE; →) ACTION2NEAREST (SOUP;RIGHT)	ACTION2NEAREST (ONION;INTERACT) ACTION2NEAREST (SERVICE; *↓*) ACTION2NEAREST (SOUP; *↓*) POT_STATE (POT_0_; *¬*STARTED)	–

At each stage, it responds with what action it would do in the state and how it believes the state could change (both added and removed predicates after applying the action). The first row includes added predicates, and the second row includes removed predicates. The header row represents (action and *I*_*d*_(*s*′)).

Algorithm 3 **How do you plan to fulfill d from s?**

**Procedure** how(*d*, *s*, *PG*)

 *current* ← *s*

 **if** *s*⊧*d* **then** ⊳ state can fulfill desire

 **return** *A*_*d*_ ⊳ return actions that fulfill the desire

 
**end if**

 *s*′ ← *argmax*_*s*′,*a*_∈_*Succ*(*s*)_*I*_*d*_(*s*′) ⊳ maximum intention possible future state and action

 **return** cat(*a*,*s*′,how(*d*,*s*′,*PG*)

**end procedure**

Algorithm 4 **Stochastic how do you plan to fulfill d from s?**

**Procedure** how_stochastic(*d*, *s*, *C*, *PG*)

 *current* ← *s*

 **if** *s*⊧*d* **then** ⊳ state can fulfill desire

 **return** *A*_*d*_, *Success* ⊳ return actions that fulfill the desire

 
**end if**

 **if** *I*_*d*_(*s*′) < *C* **then** ⊳ intention is no longer attributed in this state, it is below the commitment threshold

 **return** *Failure*

 
**end if**

 *s*′, *a* ∼ *P*(*s*′, *a*|*s*)

 **return** cat(*a*,*s*′,how_stochastic(*d*,*s*′,*C*,*PG*)

 
**end procedure**

This algorithm provides a plausible path but does not account for setbacks or alternatives and is thus only partial. Algorithm 4 complements this by considering instead randomly sampled state successors from *P*(*s*′, *a*|*s*), recording multiple paths and classifying them between success and failure, where the former is an arrival at a state such that the action can be fulfilled and the latter is an arrival at some state where the intention is no longer attributed (i.e., falls below the commitment threshold).

Although the questions of “What is the intention?” and “How is it achieved?” are enough to explain the reasons for having intentions, answering for agent “behavior” is intrinsically tied to the choice of actions taken and, therefore, must also account for the action perspective. To do this, it is necessary to answer the third question: why an action is taken. A way to answer is to consider the possible effects an action, *a*, will have in a particular state, *s*, grounded in increases of intention that motivate the change. Actions can be broken down into unintentional and intentional. This paper defines the latter as “actions that help support further one (attributed) intention,” which means it increases the odds of it succeeding. This means an increase in *I*_*d*_(*s*) for some *d* (that is attributed in the current state) and some future state. Note that, while the sentence is phrased as “why is the action taken?” this definition allows one to reply in terms of “why would the action be taken?” and can answer about hypothetical action-taking scenarios.

This definition should take into account how intention will be modified, which depends both on the action and the future state. This could be computed as the expected intention increase from taking an action (i.e., weighted average based on *P*(*s*′|*a*, *s*)). However, this would not account for risky actions or gambling behavior. For example, a plausible explanation for participating in a lottery would be the hope of winning money. Still, the probability of such an event is low, and the expected return in money is negative. An action that can further an intention may also hinder it depending on the following state it achieves (e.g., winning or losing). Instead, the interpretation of this answer benefits from considering not just expected increases but also possible ones.

The answering algorithm is described as follows.

• If no attributed desire exists in the state, then the action is apparently unintentional from the point of view of the PG and considered desires.
• If there are attributed desires and some of them have a positive expected intention increase when executing the action, then these intentions are a sufficient explanation for the action. The expected intention increase can be computed as $E_{P\left(s^{\prime }|a,s\right)}I_{d}\left(s^{\prime }\right)-I_{d}\left(s\right)=\sum_{s^{\prime }}P\left(s^{\prime }|a,s\right)*I_{d}\left(s^{\prime }\right)-I_{d}\left(s\right)$, where $E_{P\left(x\right)}f\left(x\right)$ is the expected value of *f*(*x*) under the probability distribution *P*(*x*).
• If there are attributed desires but none have a positive expected increase in intention, the action may be a gamble: the intention has a low probability of increasing by a given amount. The explanation includes the probability of a positive increase (*P*(*I*_*d*_(*s*′) ≥ *I*_*d*_(*s*)|*s*, *a*)) and the expected increase in such a case $\left(E_{P\left(s^{\prime }|a,s,I_{d}\left(s^{\prime }\right)\geq I_{d}\left(s\right)\right)}I_{d}\left(s^{\prime }\right)\right)$. An explainee can consider these values to gauge how likely the action was to further the intention and by how much. A probability distribution function can also be considered, showing *P*(*I*_*d*_(*s*′) − *I*_*d*_(*s*)|*s*, *a*) for visual analysis. If neither metric is acceptable for any desire, then behavior can also be considered unintentional from the point of view of the PG and considered desires.

We find that the last behavior’s reply is quite complex and not suited for most explainees. However, this behavior frequently happens when analyzing RL agents and can be useful in the context of debugging the agent. Our hypothesis regarding this frequency is that there may exist vestigial exploration behavior (i.e., trying *a priori* non-optimal actions to test if there are unexplored possibilities that are better than the current optima).

An important caveat of the method has to do with considering counterfactual explanations. For example, when questioning an agent’s behavior, an explainee with preconceptions over optimal behavior would ask, “Why did you not choose action *a*′ at state *s* (which I believe to be optimal)?” This question can be addressed via asking it in positive (i.e., why is *a*′ taken?) and comparing with the same answer but for the other action. This leaves a degree of interpretation when comparing results. Beyond that, it is possible that, when asking, “Why is *a*′ taken in state *s*?”, it is impossible to answer: such is the case if action *a*′ is never taken in *s*, and as such, the consequences and effects on intentions are unknown. This is an inherent limitation of working with observational models: answering counterfactual explanations requires more than statistical, associative knowledge and requires interventions in the environment (i.e., it would require knowing *P*(*s*′|*do*(*a*), *s*), as *P*(*s*′|*a*, *s*) is undefined if *P*(*a*|*s*) = 0). These are currently outside the grasp of PGs. We note that the availability (or lack thereof) of this information could be used as a mechanism to condition agent behavior (e.g., be used as a form of curiosity or intrinsic motivation).

Finally, to find EF explanations, a potential avenue would be to answer queries such as “When is an intention for *d* manifested?” or “What properties does a state *s* need to hold so that the agent commits to desire *d*?” As of now, intentions are computed from future states, but since current state properties determine the probability of reaching future states, there should be a causal relationship between state properties and manifested intentions. For example, an agent may manifest the intention to deliver whenever a pot is in the finished state: this could be used as a rule of thumb to predict intentions independently of knowing future states.

In summary, we have presented three main explainability mechanisms (questions and ways to produce answers) that can be built on the basis of intentions and PG transition knowledge. The purpose of the questions is to provide explanations of the RE form, the most common one. These algorithms are shown to produce explanations in pseudo-natural language and are structured in a way such that it is possible to process them into natural language explanations or use their structured, computer-understandable responses for other downstream tasks (such as coordination between agents). Compared to explanations such as the ones provided by SHAP or LIME, our technique offers local explanations (tied to a state) that are much more interpretable and complete. Furthermore, SHAP and LIME are restricted to explaining behavior one action at a time, while the questions presented in this section can help predict the long-term behavior of the agent. These reasons, as well as the fact that SHAP/LIME and IPG explanations can be seen as complementary, justify the usage of an IPG when the time and resources are available. The use case permits the usage of IPGs (see limitations for use cases where it is not possible).

### Metrics

Having presented several heuristics and considerations for PG design, the need to validate the model arises. Achieving the desired balance between reliability and interpretability cannot be a blind task. Much like the intended explanations, the outcome of the design process should be quantitatively analyzed to validate or give feedback on the process of designing a PG of the appropriate characteristics for the problem.

In this section, we propose computable metrics that allow the agent designer to assess and quantify the performance and effectiveness of the explanations produced by models built with our proposed pipeline. As metrics, they define metric spaces in which explanations can be quantitatively compared and ranked, as well as a distance function that allows, for example, expressing, “How effective is an explanation compared to another one?”

Given that the proposed pipeline works in two stages (first constructing a PG and then proposing desires and intentions), the metrics in this section are split depending on which specific part of the pipeline it makes sense to apply them to.

Static metrics can be seen in the literature,^22^^,^^85^ which take the PG as a probabilistic graphical model (PGM) and analyze its properties statically. Although widely used and intuitive, these metrics have inherent weaknesses. One source of this is that no information on the criticality of a decision in a state is available on a PG, and as such, these works tend to use surrogate functions to estimate criticality. We present the limitations of static analysis in a toy experiment in are static metrics not enough?: the traffic light thought experiment.

However, such a problem can be solved by introducing desires and metrics that leverage their information to compute the reliability and interpretability of explanations. As these metrics require a set of explainee-defined desires (which can be created iteratively), the guides they provide in early stages may be biased to a suboptimal representation. As such, we propose relying on static and intention metrics, leaning more on the latter as the PG is refined.

#### Static metrics

Static metrics analyze the graph’s properties regardless of intentions and desires. This allows for an idea of the variability of the expected agent behavior in different scenarios, which can be helpful to pick the best state representation for the PG and compare several ones. We consider three approaches to the task, each evaluating different but relevant points: entropy, behavioral similarity, and trajectory likelihood.

Entropy is one of the most natural ways of evaluating how informative the PG model is: if knowing the current state unequivocally determines the following action and state, then the PG is perfect, the explanations are entirely reliable, and a policy derived from it could replace the original agent. This occurs mainly in toy cases, but entropy helps quantify proximity to the ideal state.

For PGs, state entropy is computed as follows:

(Equation 1) $$H\left(s\right)=-\sum_{s^{\prime },a\in \left\{s^{\prime },a:P\left(s^{\prime },a|s\right)\neq 0\right\}}P\left(s^{\prime },a|s\right)*log_{2}P\left(s^{\prime },a|s\right)\text{.}$$

This metric can be understood as the expected number of bits required to encode the immediate future of the node: the lower the metric, the less uncertainty there is about the agent’s and environment’s behavior. The future of the node may be further decomposed into two factors: action entropy H_*a*_(*s*) (Equation 2), and future state (or world) entropy H_*w*_(*s*) (Equation 3), holding that H(*s*) = H_*a*_(*s*) + H_*w*_(*s*).

(Equation 2) $$H_{a}\left(s\right)=-\sum_{a\in \left\{a:P\left(a|s\right)\neq 0\right\}}P\left(a|s\right)*log_{2}P\left(a|s\right)$$

(Equation 3) $$H_{w}\left(s\right)=-\sum_{a\in \left\{a:P\left(a|s\right)\neq 0\right\}}P\left(a|s\right)*\sum_{s^{\prime }\in \left\{s^{\prime }:P\left(s^{\prime }|s,a\right)\neq 0\right\}}P\left(s^{\prime }|s,a\right)*log_{2}P\left(s^{\prime }|s,a\right)$$

The decomposition of entropy in two parts shows a key insight on the balance for creating a PG: a low number of different discretized states results in fewer possibilities for *P*(*s*′|*s*, *a*) and likely a lower H_*w*_(*s*), but at the same time, it is likely that a state *s* determines the following action perfectly by *P*(*a*|*s*) and thus lowers H_*a*_(*s*). This equilibrium is also present on the reliability and interpretability side: the more states there are, the more difficult it is to understand agent behavior, as one must shift to local state regions to analyze graphs that are too large. However, overly simple graphs with few nodes lead to greater action uncertainty, reducing reliability. It should also be considered that the larger the PG, the more agent observations should be taken to lower the variance of estimations of *P*(*s*′|*s*, *a*), or the resulting graph will not be reliable, even despite entropy computations. These entropy metrics can be extended to the entire graph by computing the expected value ($E\left(H_{x}\left(s\right)\right)=\sum_{s}P\left(s\right)*H_{x}\left(s\right)$, for H(*s*), H_*a*_(*s*), and H_*w*_(*s*)).

In the literature, the mean of entropies has also been observed^22^ by not accounting for *P*(*s*). This can be a desirable change, especially given that, for some problems, taking specific actions may only be critical in certain unlikely states, whereas there are states in which any action is comparable and action selection matters less. The lack of consideration for state criticality (i.e., when is it paramount to capture agent behavior to give correct explanations?) is a limitation of entropy. We discuss this in the are static metrics not enough?: the traffic light thought experiment section, with intention metrics used to support it.

Besides intention metrics, another static metric can be used to, if not verify, measure how faithfully the PG considers state criticality when given access to a reward (for the case of RL agents). An action-selection policy *a*←*π*^*PG*^(s) can be built by sampling from *a*∼*P*(*a*|*disc*(s)), thus allowing the creation of agent surrogates. If the PG creator can test agents in the environment and they know the original reward function, they may compare their performance, as in prior work.^24^^,^^85^ The surrogate agent reward decay (or reward decay, Δ*R*(*T*)) is computed as the difference in expected reward between the two policies (in episodes of length *T*):

(Equation 4) $$\Delta R\left(T\right)=E\left[\sum_{t=1}^{T}R\left(s_{t},\pi \left(s_{t}\right),s_{t+1}\right)\right]-E\left[\sum_{t=1}^{T}R\left(s_{t},\pi^{PG}\left(s_{t}\right),s_{t+1}\right)\right]\text{.}$$

This can be trivially extended to cases where performance is held out to the end of the episode as $\Delta R=E\left[R\left(\pi \right)\right]-E\left[R\left(\hat{\pi }\right)\right]$.

The intuition behind this metric is that the relevant predicates for explaining the agent’s actions are also relevant for acting. As such, the reward decay obtained by simplifying the agent can be linked to the decay in the reliability of our explanations. More importantly, this metric reduces the relevance of PG misrepresenting the agent in states that have no influence on reward and informs the designer better on whether the *important* behavior of the agent is well represented.

A near-zero decay in Δ*R*(*T*) indicates similar performance between the original and surrogate agents. However, the fact that this value can sometimes be negative (i.e., the PG agent obtains better rewards on average than the original agent) could mean that the PG and original agent capture different policies, even when this metric is high. Although potentially desirable from a performance standpoint, this casts doubt on whether the PG provides reliable explanations of the agent, as it has captured something different.

To summarize the utility of these metrics, they can be boiled down to their usage. Entropy can be used to understand how much information a PG provides in terms of predicting states and actions: if, in a real state, it takes *N* bits to present the information of which is the next state and action, this is reduced to H bits. The components of the entropy (agent and world entropies) can be used to understand the trade-off between how much information the PG provides with respect to the next state or the action. This is contingent on the expressivity of the discretizer and can be used to pick accordingly (i.e., for high world entropy and low agent entropy, it is suggested to simplify the discretizer, and vice versa). Furthermore, when the PG designer has access to the environment and reward function, they may compute reward decay to further understand how well the PG captures agent behavior in states that have an influence on the performance of the agent. An agent designer of PG should use these metrics as feedback for future iterations of their discretizer.

#### Intention metrics

To gauge the explainability of the PG with intentions, one should consider two things: how likely is it that *s* (the state analyzed) can be said to hold an intention *I*_*d*_, and thus *s* can be used to explain, and how likely is it that, if the *PG* claims an intention for a state, such intention holds?

As proposed previously, intentions should be attributed to a state only once they exceed a certain threshold: the commitment threshold *C* > 0. This is because even if the agent may have some non-zero probability of achieving a desire in a state, an explanation claiming that the agent has such an intention is not desirable if such probability is very low, making a cutoff necessary to reduce human bias. We define the set *S*(*I*_*d*_) = {*s* ∈ *S*|*I*_*d*_(*s*) > *C*} as the set of states where the agent is attributed as having the intention *I*_*d*_. In addition, we also consider the set *S*(*I*) = {*s* ∈ *S*|∃*d* ∈ *D*: *I*_*d*_(*s*) > *C*}, that is, the set in which the agent is attributed as having any of the considered desires as its intention.

By classifying states as either having or lacking an intention, we can extend the probabilities used to answer the questions above.

(1) Probability of intention attribution *P*(*s* ∈ *S*(*I*_*d*_)) is the probability that, at any point of observation, the agent is in a state *s* that fulfills *I*_*d*_(*s*) > *C.*
(2) Expected intention $E_{s\in S\left(I_{d}\right)}\left(I_{d}\left(s\right)\right)$ is the probability that, once attributed, an intention is going to be fulfilled. It is computed as $E_{s\in S\left(I_{d}\right)}\left(I_{d}\left(s\right)\right)=\sum_{s\in S\left(I_{d}\right)}I_{d}\left(s\right)*P\left(s\right)/P\left(s\in S\left(I_{d}\right)\right)$.

The first metric estimates the interpretability of agent behavior (the maxim of manner): the less likely it is that the agent has no attributed intention in the state, the fewer times we will have no answer for why it is acting. The lower the commitment threshold, the larger the attributed intention probability. For the case of *S*(*I*_*d*_), this score can also be increased by introducing more desires to check.

The second metric is an estimation of the reliability of an explanation (the maxim of quality). It computes how likely it is that an explanation of why it did something (the cause) did not result in it (the consequent) being fulfilled.

While both metrics can theoretically reach 1 (a perfect state), real-world scenarios rarely allow this. On one hand, for a sufficiently low *C* and enough desires considered, it is likely possible to reach maximum probability of intention attribution (i.e., always being able to attribute why) but at the cost of being wrong several times. On the other hand, even with a high *C* value, it is likely that an agent that has an intention to achieve something may fail due to unexpected environmental changes.

#### Are static metrics not enough?: The traffic light thought experiment

Static metrics and prior literature^22^ have a key limitation: they assume that action-selection uncertainty impacts the value of explanations equally across all states. However, in most real-world scenarios, it is seldom the case that behavioral certainty is critical. In other words, in most states, an action can be liberally chosen. Using *AD* as an example, a car in an empty highway can liberally switch lanes or accelerate. If instead there was a car in its left side, it is critical that it does not switch lane to the left (to avoid a crash).

Let us define a critical state-action as a transition (i.e., doing *a* in *s*) that is important in an environment (and to an explainee). This can be due to some reward (positive or negative), design principles, or any reason why the agent’s stance toward doing (or not doing) *a* in *s* is important. By definition, an algorithm that provides explanations that faithfully account for *P*(*a*|*s*) in critical state-action is more trustworthy than one that does not, even if the latter provides more reliable or interpretable explanations in other situations.

The lack of consideration for these situations offers a biased estimation of *PG* adequacy, given our lack of context on the criticality of the states.

Proposition 1: presented with two surrogate *P*(*a*|*s*) models *A* and *B*, where model *A* outperforms *B* in terms of expected agent entropy, it can be the case that model *A* misrepresents critical state-action pairs more than model *B*, and thus model *B* is more trustworthy than model *A*.

To prove this point, we present a minimal example where this can happen: the traffic light environment and agent. In this environment, there are three (non-discretized) states, the traffic light is red (*R*), yellow (*Y*), or green (*G*), in which the agent can take four actions (going left, right, down, or up). The only rewarded transitions are going up on *G*, which gives a positive reward, and going up on *R*, which gives a negative reward. This defines these two states as critical from an external perspective: a PG that misrepresents the action probability of those actions in those states is misleading. Regarding transition probabilities, to simplify computation, neither the current state nor the action choice affects the next state. The next state probability has some strong bias toward *R* or *Y* (50% and 45%), and a very low probability of going to *G* (5%).

**Figure 4:**
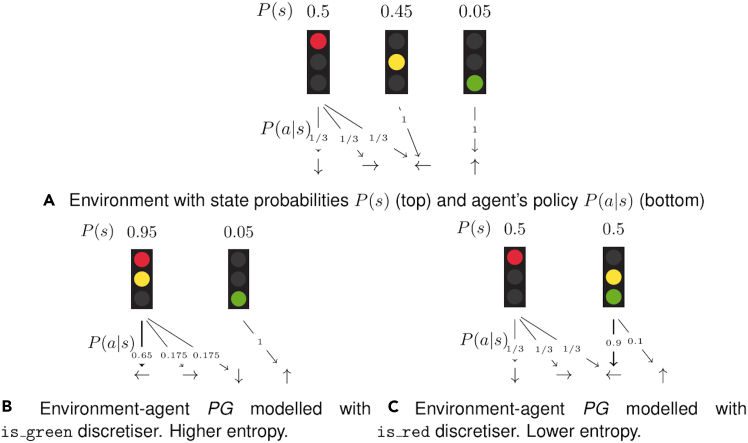
The traffic light environment and the proposed discretizers Agent observations consist of viewing the state of the traffic light as in environment (A). Discretized observations can only tell one of the states apart from the other two. Colors have been placed to distinguish between different *P*(*a*|*s*) values according to the discretizer. While is_green preserves the policy in critical state-actions as seen in environment (B), is_red severely misrepresents the policy for green states as seen in environment (C). While, according to this, is_green PG is a better discretizer, its entropy is worse than the is_red one.

Suppose now an agent that interacts with this environment as follows: in the *R* state, it uniformly samples the action between left, down, and right (33%); in *Y*, it always goes left (for some undisclosed and irrelevant reason, not tied to its design or goals); and in *G*, it always goes up. This agent has an optimal policy for this environment. Figure 4A illustrates this arrangement.

Suppose now two PGs: one with a single predicate is_green, which distinguishes the *G* state from *Y* and *R*, and one with a single predicate is_red instead. Both of these discretizers are illustrated in Figures 4B and 4C. Neither will be a perfect surrogate, as all three states have a different probability distribution over actions. The first one can represent an optimal policy as the probability of the critical action-state pairs (never goes up in red, always goes up in green) is preserved, even if it misrepresents the probability of picking any action in yellow (non-critical). The second surrogate cannot, as it *severely* misrepresents the probability of going up on green, attributing instead a 90% probability of going left. This shows that, for the purpose of giving explanations, is_green is more faithful in critical states than is_red.

This is not apparent from the perspective of static metrics. When computing the agent entropy between these agents, it becomes apparent that *H*_*a*_ is higher (less desirable) for the first case (0.89) than for the second (0.71). This happens because the relevance of the critical case gets subsumed when considering large numbers.

The probability of this happening increases with the size and complexity of the environment, but, as has been shown, it can happen in toy examples. Although removing from the entropy the weighting of states by their probability (heavily biasing toward infrequent states) or computing entropy on a subset of states found heuristically can reduce the relevance of the problem,^22^ it can become unreliable if the heuristic is mismatched to the problem. For example, when choosing critical states where *H*_*a*_ is low, the PG could misreport explainability in critical states where action entropy is large, such as the red traffic light in this example.

Instead, this problem can be avoided by modeling the environment so that desires and intentions provide the external knowledge on critical state-actions. Any metric not incorporating the needs of the explainee toward critical state-actions can be a victim of misrepresenting the trustworthiness of the model (e.g., by overestimating the relevance of a non-critical state-action, such as going left on yellow, or underestimating the relevance of an improbable but critical state, such as going up on green). In this toy example, neither discretizer can model both the desire to go up in green and the desire not to go up in red directly (either predicate is unable to speak of the other). Nonetheless, if modeling both predicates was not possible, it is still viable to formalize the desires colorfully (e.g., the desire not to go up is in any state with the possibility of being red), in which case the intention metrics would report higher reliability in the is_green PG.

### Revision pipeline

All previous metrics offer empirical, quantitative qualifiers of the designed PG and can be used to report expected performance (both from the side of reliability and interpretability). However, the quality of the metrics and the explainability extracted depend highly on the PG design, which is done with little information to start with. To address this problem, it makes sense to leverage the newly produced intentions as feedback to debug the agent, the discretizer, and desires and to improve each of them.

This can be done by analyzing intentions as they progress over time along trajectories to gather why the representation may be inaccurate and enhance it. These trajectories may be actual agent observations or can be simulated by sampling the PG if the agent cannot take new observations. We call this the revision pipeline. We show revision pipeline examples where we use these to debug some of the agents in the paper.

There are two prominent cases that can be detected and used to improve the system.

• Regions without intention: these are long sequences where no intention is manifested above the commitment threshold. The presence of such regions is trivial to find given a trajectory, a commitment threshold, and a minimum time-step length. There are two possible causes: either no agent’s desire exists that can be manifested or the explainee or designer never declared the intentions transpiring during such sequences. This kind of feedback can be addressed via observing samples of the trajectory and considering the following:
  – If there is a new possible desire that was ignored, that may be happening in this trajectory. The concise summarization of many and long trajectories into a small subset of regions without intention makes it easier to hypothesize new desires *ad hoc* to the seen situations, adding them to the IPG*.*
  – If there is an existing desire that is apparently fulfillable in this region but the IPG shows that the agent never attempts. This may indicate that the desire is not present under circumstances similar to those in the trajectory and may require modification. Alternatively, it may indicate that the agent is not rational with respect to the hypothesized desires, and this information could be used to improve the agent via fine-tuning in these situations (i.e., using these data as a signal during training to foster the fulfillment of desires when the agent is in these located states).
  – If there is no possible desire that can be fulfilled. This may be the case when there are no affordances for acting in any way and the agent is waiting. While in some situations this may be beyond the system designer’s control, there may be cases where it can be addressed by modifying the environment (or other agents in an MA system).
• Unfulfilled regions: these are sequences in which an agent was attributed with an intention but it was not fulfilled (i.e., the intention fell below the commitment threshold or it remained with high intention after an inordinate amount of time). The presence of these regions can be located when the conditions to consider a region unfulfilled are established, but finding which unfulfilled regions are meaningful to debug is best left to a case-by-case, iterative process. The causes for unfulfilled regions can be related to prioritization of a different and conflicting intention, irrational agent behavior, hidden desires, or the discretizer function not distinguishing between two different (real) states that have different (real) intentions. This kind of feedback can be addressed via observing samples of the trajectory and considering the following:
  – If there are, instead, other desires being fulfilled during the period of time. This may indicate that the desire is conflicting with others.
  – If the agent is failing to perform its task because of the environment (i.e., the agent attempts to fulfill the desire, but the necessary transitions to do so are up to the transition function of the environment). This case may suggest that the desire is hard to fulfill. In some cases, it may be possible to improve the system (environment, agent embodiment, or other agents) to increase the probability of success.
  – If the agent is instead taking irrational actions that fail to accomplish the desire. This information could be used to improve the agent via fine-tuning in these situations.

The agent’s uncertainty can be concisely presented by reporting these regions of interest. A human counterpart can analyze the regions and develop new hypotheses based on the guidelines above, such as proposing new desires, filtering the reports based on a hypothesized prioritization of desires, or hypothesizing how the policy may improve via a concrete change in behavior in a situation. How these hypotheses are implemented is up to the designer or explainee—new desires need to conform to newly developed expectations of the explainee for them to be understandable—and which changes to perform to the original agent or system depends on the desiderata of the system. In both cases, however, the revision pipeline can serve to isolate the relevant cases from more common or understandable trajectories that do not give meaningful feedback to change the system.

## Results

So far, in this paper, we have introduced the following contributions:

• A methodology for producing explanations for agents’ behavior, based on constructing PGs from the agents’ observations and discretizing the state space and a set of desires.
• Explainability questions and answers covering IPG behavior.
• Static metrics for analyzing the structure of the PG*.*
• Intention metrics, capable of measuring both the interpretability of the agents’ behavior and the reliability of the explanations produced—both in terms of attributable intentions derived from the proposed desires.
• A pipeline for interactive revision of the PGs, which automatically identifies regions of interest in the timeline of the agent’s behavior.

In these sections, we present empirical results for the application of these metrics and of the revision pipeline to the two use cases: the Overcooked-AI environment^83^ and the nuScenes^84^ driving dataset. The library for producing the PGs is *pgeon*,^96^ which is being developed by the authors, among other contributors.

The experimentation methodology can be summarized as follows:

(1) We select some training methods, and for each layout, we train specialized agents from scratch.
(2) We analyze the performance of the resulting agents.
(3) We design a set of discretizers to compare the effects on the metrics of expressing the state with or without specific predicates, and we propose a set of desires relevant to each scenario.
(4) We apply and analyze the static and intention metrics to the resulting PGs.
(5) We analyze the results of applying the revision pipeline to this environment and discuss its potential use from the perspective of an agent designer as the explainee.

### Experimental results in the Overcooked-AI scenario

All experiments for this scenario have been conducted in the Overcooked-AI environment, and the training code has been developed using Pantheon-RL (https://github.com/Stanford-ILIAD/PantheonRL). The PGs were generated by observing 1,500 episodes, with up to 400 steps per episode. The performance metrics (i.e., accumulated rewards) have been computed as the means and standard deviations of 500 episodes in random environments per agent. The hardware used was an Intel i7-5820k system with 96 GB of RAM and an Nvidia RTX 3090 GPU.

This experimentation section is structured as follows. First, the choice of the training method for each agent is presented and motivated. After that, the options for discretizing the state space and for the static metric analysis are developed. Finally, intention metrics are used to analyze each combination, and a case study is conducted with one of these and the revision pipeline to demonstrate the type of explainability that can be produced.

#### Agents used: Overcooked-AI

The agents analyzed in this paper consist of three pairs of agents that collaborate.

• Pair A (PPO agent 1 [blue] and PPO agent 2 [green]): two agents trained from scratch with PPO.^97^ These agents were used in previous work^85^ to evaluate if PGs can serve as surrogate models of an agent, which depended on the discretizer and layout. For example, in layout random_0, the blue agent’s surrogate was only able to match the original’s performance in discretizers with information on the other agent’s position: *D*3 or *D*4 (see discretizers and static metrics: Overcooked-AI).
• Pair B (human agent [green] and human-collaborating agent [blue]): a human agent trained from human trajectories exclusively and a PPO agent trained to collaborate with it. These agents were used in previous work.^23^^,^^83^ It is important to note that some behaviors learned by the PPO agent trained to collaborate with the human are suboptimal due to the lack of co-adaptation. For example, based on the experimental results shown in Figure 3, we verify that for the unident_s layout, the behavior of the human-collaborating agent is random and was not trained correctly despite its apparently high performance metrics in Table 3.

**Table 3:** Performance evaluation (mean obtained reward and its standard deviation) of the trained agent pairs

	PPO agent 1 and PPO agent 2	Human agent and human-collaborating agent	Random agent and PPO agent 2
Simple	387.87 (25.33)	251.26 (31.62)	21.55 (16.71)
random_1	266.01 (48.11)	187.19 (28.53)	36.70 (11.48)
random_3	62.5 (5.00)	81.93 (21.79)	0.53 (1.47)
unident_s	757.71 (53.03)	102.12 (28.11)	4.30 (7.30)
random_0	395.01 (54.43)	107.99 (46.45)	7.61 (6.03)

Performance evaluations are for each of the layouts listed in Figure 2. As would be the case in an RL problem, the numbers are not very informative given the prior lack of information on expected rewards, maximum reward, or general scale (unless the practitioner solves that problem manually or the environment provides it, which is seldom the case). For the case of unident_s, the human-agent pair obtains results only due to the human agent doing all the work, which is not apparent in this table and is one of the difficulties of debugging RL agents.
• Random baseline (random agent [blue] and PPO agent 2 [green]): same as pair A, but PPO agent 1 is substituted by an agent that samples actions from a uniform probability distribution (all actions have probability 20% regardless of the state). This agent is used as a baseline for comparison with the other two pairs.

For each of the five layouts, the five agent types (human agent, human-collaborating agent, PPO agent 1, PPO agent 2, and random agent) were trained from scratch, so there are a total of 25 different agents.

#### Discretizers and static metrics: Overcooked-AI

Four discretizers are tried and tested for each of the agents and environments. From 1 to 4, each is more expressive and increases complexity (and entropy). The main discretizer includes all predicates relevant to behaving in the environment, including the state of the pots and the relative positions of objects (which drastically reduce complexity). Each extension focuses on enhancing information about the other agent’s state and its actions.

Table 4 gives the full description of each discretizer. Predicate computation is done via the environments’ MediumLevelPlanner. Each variable may take only one value in a state. “held” and “held_partner” represent the object the agents are holding, where O, T, D, and S stand for the items that can be held (onion, tomato, dish, and soup, respectively). “item_pos” shows the optimal next action to get to a specific item (be it an item source or not), where ↑, ↓, ←, →, I, and S stand for the actions to reach an item (go up, down, left, right, interact, or stay). “partner_zone” refers to the cardinal direction in which the other agent is located with respect to the PG agent. Note that ↑, ↓, ←, and → are only used when the two agents are on the same horizontal or vertical axis.

**Table 4:** Variables used to describe the domain by each discretizer, where items = {O,T,D,pot,service}

	Variables (domain)
D1	held(O,T,D,S,Ø)
	pot_state(empty,waiting,cooking,finished)
	item_pos(↑,↓,←,→,I,S),∀*item* ∈ items
D2	*D*1∪{held_partner(O,T,D,S,Ø)}
D3	*D*1∪{partner_zone(↑,↗,→,↘,↓,↙,←,↖)}
D4	*D*2 ∪ *D*3

Likewise, Table 5 illustrates the static metrics for a subset of agents and layouts. The best metric per agent and layout is marked in bold. Each of the entropies behaves as expected: *H*_*w*_ (the entropy on what is the next state) always increases with the complexity of the discretizer, as there are more possible states to go to, whereas *H*_*a*_ (the entropy on what action the agent will take) has a tendency to but does not always decrease—especially in poorly performing agents. Although there exists a correlation between *H*_*a*_ and Δ*R* (the difference in reward between the surrogate and original agents), results are inconclusive given the variability of Δ*R*. The random agent (the baseline) shows that a policy independent of the predicates introduced cannot reduce the PG’s *H*_*a*_.

**Table 5:** Static metrics of the human-collaborating agent, PPO agent 1, and random agent in the simple, random_0, and unident_s layouts

Layout	Agent	*D*	*H*	*H*_*a*_	*H*_*w*_	Mean Δ*R*
Simple	human-collaborating agent	1	**1.98**	1.46	**0.52**	−60.96
		2	2.15	1.41	0.74	−34.66
		3	2.10	1.38	0.72	−25.26
		4	2.21	**1.31**	0.90	**−7.36**
	PPO agent 1	1	**2.13**	1.68	**0.44**	−19.39
		2	2.40	1.62	0.78	−15.51
		3	2.47	1.50	0.98	−7.76
		4	2.45	**1.43**	1.02	**−3.88**
	Random agent	1	**3.37**	2.57	**0.80**	0.69
		2	3.39	2.56	0.83	−0.17
		3	3.60	2.56	1.05	−0.05
		4	3.56	**2.54**	1.02	**0.98**
random_0	human-collaborating agent	1	**2.17**	1.70	**0.48**	−107.99
		2	2.25	1.57	0.68	−107.99
		3	2.44	1.65	0.79	0.61
		4	2.40	**1.49**	0.91	**8.61**
	PPO agent 1	1	**1.54**	1.03	**0.50**	−19.75
		2	1.60	0.98	0.62	−15.80
		3	1.65	0.98	0.67	**−11.85**
		4	1.68	**0.93**	0.75	−19.75
	random agent	1	**2.96**	2.58	**0.38**	−0.23
		2	2.97	**2.57**	0.40	**−0.04**
		3	2.97	**2.57**	0.39	−0.76
		4	2.97	**2.57**	0.40	−0.07
unident_s	human-collaborating agent	1	**2.14**	1.86	**0.27**	−13.02
		2	2.26	1.76	0.49	**−10.82**
		3	2.47	1.85	0.62	−13.22
		4	2.49	**1.74**	0.76	−13.72
	PPO agent 1	1	**1.37**	0.90	**0.47**	−7.58
		2	1.65	0.88	0.77	−7.58
		3	1.82	0.86	0.96	−7.58
		4	1.89	**0.84**	1.06	−7.58
	random agent	1	**3.15**	2.58	**0.57**	−0.10
		2	3.16	2.58	0.58	−0.23
		3	3.56	**2.57**	0.98	−0.05
		4	3.52	**2.57**	1.96	**0.57**

*H*, *H*_*a*_, and *H*_*w*_ correspond to entropy static metrics (lower is better), while Δ*R* is the mean difference in reward between the surrogate and the original agent (higher is better). The best performance value for each combination of agent and layout is marked in bold.

The results indicate a complex trade-off between the reliability and interpretability of the PGs. There is no clear winner in any category. Still, ultimately, the representations with a richer—and therefore more complex—set of predicates represent the agent’s behavior more faithfully in the general case (as illustrated by the mean Δ*R*). Larger graphs mean more information for the agent’s actions, but as can be seen from the human-collaborating agent in unident_s, if the agent does not perform well (or ignores the added information), *H*_*a*_ may not decrease enough to warrant its use in the presence of a drastic increase in *H*_*w*_. In the case of a tie, Δ*R* can be a *reasonable* estimate of whether the PG correctly captures agent behavior. Thus, the explainability extracted from it is either reliable enough or should be discarded as the surrogate model is not capturing the behavior of the original agent where it matters most (i.e., performance). These findings motivate future work aimed at experimentally evaluating whether alternative training strategies can address the observed shortcomings.

#### Intention metrics: Overcooked-AI

Static metrics offer direct, unbiased insight into the structure of the PGs. When the differences are significant enough, agents can use them to reliably tell which families of discrete options trump the rest. However, the relationship between static metrics and PG adequacy is challenging to understand. When the difference in metrics between the two options is too small, it becomes easier to evaluate the methods from the perspective of the maxims of communication or the correctness of explanations that the PG may produce.

To better evaluate the quality of explanations, it is necessary to gain insights into the agent’s goals and objectives, which, in this paper, requires external (human) information. A formalization of desires is introduced, allowing the PG to manifest beliefs over beneficial agent behavior. By extending desires into the past, it becomes possible to evaluate what possible beneficial behavior the agent is likely to manifest in the future (i.e., what intentions it holds). However, external insights into the agents’ goals may be biased or outright wrong. As such, it becomes necessary to evaluate the adequacy of the PG and the human-hypothesized agent’s desires. In exchange for this added complexity, it becomes possible to directly evaluate the trade-off between the reliability and interpretability of the agent’s behavior.

Given a PG and a desire *d*, informally, the interpretability of behavior over a desire *d* $\left(I_{d}\right)$ is defined as the proportion of time in which the agent is found in a state where it has an intention to do *d* attributed (i.e., the probability of being in a state with *I*_*d*_ above commitment threshold):

(Equation 5) $$I_{d}=E_{P\left(s\right)}\left(\left[s\in S_{d}\right]\right)=\sum_{s\in S_{d}}P\left(s\right)\text{,}$$

where [*s* ∈ *S*_*d*_] is the Iverson bracket (i.e., it is 1 if the condition within is true and 0 otherwise). This can be generalized to overall interpretability (i.e., I, the probability of being able to interpret its behavior with an intention) by computing the probability that the agent is found in a state where it is attributed any one intention:

(Equation 6) $$I=E_{P\left(s\right)}\left(\left[\exists d:s\in S_{d}\right]\right)=\sum_{s}\left[\exists d:s\in S_{d}\right]P\left(s\right)\text{.}$$

The reliability of the explanations generated using a PG, with respect to a desire *d* (denoted $R_{d}$), is defined as the expected probability that a state attributed with the intention to achieve *d* actually results in the fulfillment of *d*. By definition, the probability for a single state is equal to the intention value *I*_*d*_(*s*), so this is the expected value of the intention:

(Equation 7) $$R_{d}=E_{P\left(s|s\in S_{d}\right)}\left(I_{d}\left(s\right)\right)=\frac{\sum_{s\in S_{d}}P\left(s\right)*I_{d}\left(s\right)}{I_{d}}\text{.}$$

This can once again be generalized (R) by taking the *max*_*d*_*I*_*d*_(*s*) for any state with any intention attributed:

(Equation 8) $$R=E_{P\left(s|\exists d:s\in S_{d}\right)}\left(max_{d}I_{d}\left(s\right)\right)=\frac{\sum_{s}\left[\exists d:s\in S_{d}\right]P\left(s\right)*max_{d}I_{d}\left(s\right)}{I}\text{.}$$

**Figure 5:**
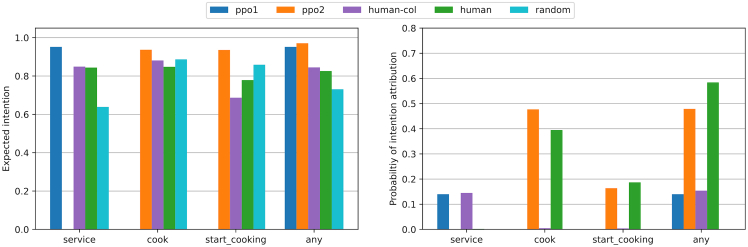
Intention metrics in the simple layout Expected intention (left) and probability of intention attribution (right) for the simple layout, using discretizer 1.

**Figure 6:**
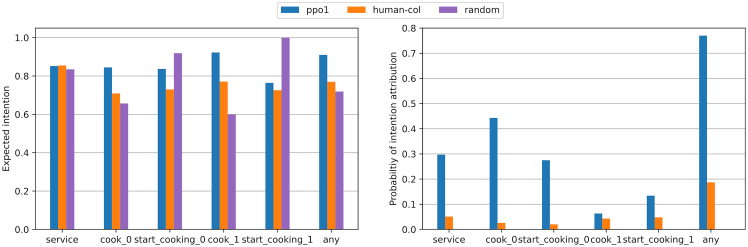
Intention metrics in the random_0 layout Expected intention (left) and probability of intention attribution (right) for the random_0 layout, using discretizer 1. Note that both PPO agent 2 and the human agent displayed no attributed nor expected intention in this layout.

Figures 5 and 6 show these metrics for the five agents in the same layouts (simple and random_0) and a single commit threshold. This information can be used to gauge how likely the method is to provide satisfying explanations to the explainee.

In Figure 5, collaboration and specialization can be seen (in each pair, one agent specializes in serving and another in cooking). Both PPO agent 1 and human-collaborating agent specialize in delivering soup, whereas PPO agent 2 and the human agent specialize in cooking. With a 0.5 commitment threshold, expected intention fulfillment is very high for all cases. Still, overall agent interpretability is low (15% of the time) for agents specializing in delivering soup (as they spend most of the time apparently idle). The random agent appears to have high reliability in fulfilling intentions: this corresponds to states in which executing random actions eventually leads to fulfilling a desire. These states happen with a probability of <0.1%.

Note that in Figure 6, it can be seen that, with a 0.5 commitment threshold, PPO agent 1 has remarkably high metrics: 77% of the time, there is an attributed intention that gets fulfilled with 91% certainty. The lack of access to the pot and service zone for PPO agent 2 and the human agent means that their behavior cannot be interpreted as reflecting these desires, and new ones should be considered (such as placing an onion or a plate on the counter). Much like before, the random agent has high reliability. Given the layout’s constrained space, it may be easier to fulfill desires at random, but the probability of manifesting intentions is low.

Therefore, each desire can be analyzed separately, and the hypothesized desires can be verified. If there is no commitment threshold at which the two metrics are decently high, it becomes apparent that the desires do not capture the agent’s behavior. This can be because either the agent did not train correctly (making the hypothesized desires unattainable) or the agent is targeting a different set of desires. This last case is apparent in Figure 6: both PPO agent 2 and the human agent had no access to the pot or the service, and thus their desires were never fulfilled.

**Figure 7:**
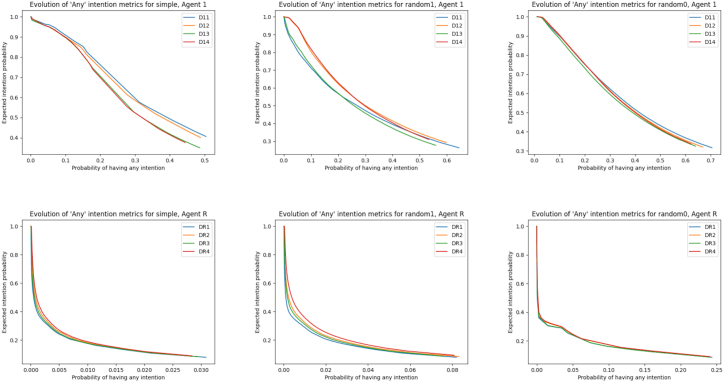
ROC curves for intention attribution and expected intention progression Probability of intention attribution (interpretability) and expected intention (reliability) progression as the commitment threshold changes (highest on the top left and lowest on the bottom right) for all 4 discretizers and the human-collaborating agent (row 1) and random agent (row 2). Points closer to the top right corner stand for preferable commitment thresholds. An explainee may, however, prefer a discretizer with a low probability of attribution if it means higher expected intention (i.e., up), or vice versa (i.e., right). Human-collaborating agents show a somewhat flat slope, with the trade-off being somewhat linear, while the random agent shows that there is no possibility for reliable attributions (i.e., up) that are even slightly probable (i.e., right).

Analyzing each of these metrics to pick the best discretizer and commitment threshold can be challenging. To simplify the process, a receiver operating characteristic (ROC)-like curve is proposed, plotting the interpretability against the reliability. In doing so, the fitness of each discretizer is displayed, and the designer can choose a better discretizer depending on the desired interpretability-reliability trade-off. In Figure 7, we can see that, for the human agent, the discretizers are mostly similar. In random_1, discretizers two and four have considerably higher expected intention probability while maintaining 10% probability of intention attribution (the content of the other agent’s hands helping predict agent behavior); this corresponds to a high commitment threshold. Meanwhile, as should be expected, a randomly acting agent cannot be explained whatsoever in terms of these desires and intentions, which correspond to a very small area under the curve (save for random_0, in which the state space is small enough that the agent does fulfill these desires with some frequency).

#### Revision pipeline example: Overcooked-AI

The intention metrics analyzed indicate that the agent behaves as desired (or as hypothesized) in most cases, except for the random agent and some agents in unident_s. However, knowing what proportion of the graph (and, thus, behavior) is explainable is insufficient to bridge the gap and discard inexplicable behavior. Instead of manually inspecting all possible states in the graph where the agent is attributed no intention, we apply the revision pipeline.

**Figure 8:**
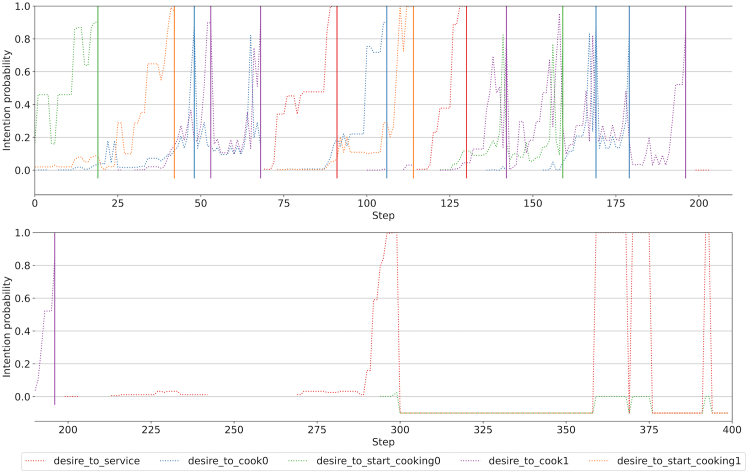
Progression of intention during one trajectory of a human-collaborating agent in the random_0 environment through time Note that for clarity, the graph has been split into two rows. Intention values at each timestep are marked with dotted lines and desire completion with vertical solid lines. Regions with an intention lower than 0 mark that the agent is in an unseen state by the PG. Each color represents a desire: red for service, blue and purple for cooking, and green and orange for starting to cook (in each pot).

To exemplify this, we choose an agent-layout pair: human-collaborating agent and random_0. The trained agent is run in the environment, during which all states (and their corresponding discretized versions) are recorded, creating a new trajectory. The progression of intentions over time is then plotted based on these states. Figure 8 shows a particularly interesting example plot for a 400-step trajectory, showing both kinds of regions of interest and a (very) infrequent case of observing new states not in the PG. The example shows three regions of interest.

The first two regions (time steps 40–70 and 125–175) are unfulfilled regions, stemming from the presence of two contradictory intentions: once given an onion, it can be put in pot 0 or pot 1. The PG information for this case is not enough to distinguish which of the two it will put the onion in. Furthermore, the selection appears to be random and irrational: the agent fills both pots concurrently and in no clear order, despite the clear benefits of filling one completely first and parallelizing the wait on the first pot with filling the second pot. The representation of the behavior of the IPG is consistent with that of the original agent, so it is correct. No apparent prioritization appears between the two desires either. This feedback could instead be used to improve the original agent (e.g., rewarding filling the fullest pot or penalizing the fulfillment of desires of pot 1 if predicate pot0_state(waiting) is true, i.e., pot 0 has some but not enough onions within). It can also be used to modify the IPG by aggregating the two desires: a desire to put an onion in either pot. Whether this change improves interpretability or reduces it because it is important to the explainee to distinguish in which pot the onion will be placed, is dependent on the explainee.

The third region (time steps 200–280) is a region without intention. The algorithm can provide no telic explanation. Observing the agent behavior in the environment or checking the predicates of states corresponding to this region reveals the reason: in this region, the agent cannot fulfill any desire, as it relies on the human agent passing a plate over the counter, which it does not do until step 280. There is nothing that the analyzed agent can do to increase its intention. The small bumps around time steps 230 and 270 correspond to the human agent getting close to the source of plates, yet it instead goes to fill more counters with onions (despite both pots being full). Again, the IPG is consistent with the original agent. This feedback could be used to modify the human agent. Still, given that the goal of this agent is to simulate human behavior, it seems reasonable to allow it to act irrationally and annoy the other agent with onions.

The fourth region (time steps 300–400) is an unfulfilled region. The agent is in a position to deliver soup: it is in the tile next to the service and it has soup. However, the agent does not serve soup despite having a very high intention. Furthermore, some of the observations in this region were discretized to previously unobserved by (and thus missing from) the PG. This situation is markedly a behavior outlier, which we justify at the end of this paragraph. While observing the agent next to the service and with soup is common, the key difference lies in the presence of an onion on the counter to its left, placed there by the human agent. This difference is enough to confuse the PG into thinking this is a new situation. Furthermore, it also generates confusion in the human-collaborating agent. The agent’s behavior alternates between interacting with the tile holding the onion and changing the direction it is facing. This feedback allows us to hypothesize what is going on: we believe that, during training, the agent was never in a position to hold soup while they could also grab an onion from the bottom-most counter. The agent appears to get stuck attempting to pick an onion from the over-the-counter display despite already holding soup; most likely, the agent learned that keeping the counter between agents empty (particularly of onions) is crucial for obtaining a reward, as plates cannot be passed over if the counter is full of onions. This complexity will pique the curiosity of researchers and developers, encouraging them to delve deeper into the agent’s behavior. In this case, the IPG does not represent agent behavior as it arrives at new states. This particular case shows that the PG was not trained with enough data with regard to this specific situation, and neither was the original agent. Furthermore, this behavior appears to be caused by the previous issue. Since the other agent filled all counters with onions instead of providing the plate, the agent finds itself in a new situation and is unable to act rationally, and the IPG suffers the same problem. Increasing the number of observations in this situation is recommended for both the original agents’ training and the IPG.

### Experimental results in the autonomous driving scenario

The experiments for this scenario have been conducted on the nuScenes “full” dataset (https://www.nuscenes.org/nuscenes). The PGs have been generated exclusively from scenes with available annotations of surrounding objects relative to the vehicle (training-validation set) and have been excluded from those exhibiting erroneous or missing data. As a result, the final dataset comprises 830 driving trajectories, each consisting of approximately 40 frames (states) per trajectory.

This experimentation section is organized as follows: the procedure for converting driving scenes from frame sequences into state-action trajectories is detailed. We propose different state discretizers and analyze the static metrics from the resulting PGs. The definitions of the desires hypothesized to influence the driver behavior are formulated and evaluated through intention metrics. Finally, we present a thorough analysis of a driving scene to illustrate the explanatory power of the revision pipeline.

#### Dataset preprocessing: Autonomous driving

To explain the driving agent’s decisions, each scene is represented as a sequence of frames and converted into a state-action trajectory. For each frame, the corresponding state is defined by extracting information about the vehicle’s dynamics, including position, velocity, acceleration, yaw angle, steering angle, and data about surrounding traffic participants (e.g., other vehicles and pedestrians) and static road objects (e.g., traffic cones) detected by the vehicle’s front camera. Information about nearby traffic elements includes their position, category (e.g., human, vehicle, or animal), visibility from the agent, and, when available, activity status (e.g., moving or parked).

Since nuScenes does not provide labels for actions executed by the driver, we design a threshold-based heuristic to annotate actions between states based on the values of velocity (*v*), acceleration (*a*), and steering angle (*δ*).

We consider a set of 10 actions, labeled according to the following conditions:

Action idle, if velocity and acceleration values are lower than the positive thresholds *ϵ*_*v*_ and *ϵ*_*a*_:

$$v<\epsilon_{v}\wedge \left|a\right|<\epsilon_{a}\text{.}$$

Action gas, if velocity and acceleration values are higher than the thresholds *ϵ*_*v*_ and *ϵ*_*a*_:

$$v>\epsilon_{v}\wedge a>\epsilon_{a}\text{.}$$

Action brake, if the velocity is higher than *ϵ*_*v*_ and the acceleration is lower than −*ϵ*_*a*_:

$$v>\epsilon_{v}\wedge a<-\epsilon_{a}\text{.}$$

Action TurnLeft, if the velocity is higher than *ϵ*_*v*_ and the steering angle is higher than the positive threshold *ϵ*_*s*_:

$$v>\epsilon_{v}\wedge \delta >\epsilon_{s}\text{.}$$

Action TurnRight if the velocity is higher than *ϵ*_*v*_ and the steering angle is lower than −*ϵ*_*s*_:

$$v>\epsilon_{v}\wedge \delta <-\epsilon_{s}\text{.}$$

Action GoStraight, which is the default action if none of the above conditions apply.

Compound actions (i.e., gas/brake + TurnRight/TurnLeft) are assigned if both motion and steering conditions are satisfied.

#### Discretizers and static metrics: Autonomous driving

Three categories of discretizers are proposed, each of increasing complexity, resulting in a total of six discretizers. The base category, *D*_0_, consists of predicates hypothesized to be the most relevant for elucidating the vehicle’s behavior. Categories *D*_1_ and *D*_2_ extend the predicate set defined in *D*_0_ by incorporating supplementary predicates that increase informational capacity but whose relevance is subject to question.

Table 6 gives a description of each discretizer. The predicates are derived from the vehicle’s state, observations of surrounding traffic participants, and data extracted from the nuScenes map, and each predicate can assume one possible value.

• “velocity” represents the vehicle’s velocity.
• “steering” represents the vehicle’s steering angle.
• “lane_position” indicates the vehicle’s position on the lane it occupies. It is categorized as aligned if the vehicle travels in the same direction as the lane, opposite if it is moving against the lane direction, center if it is positioned on a lane or road divider, and Ø if it is outside the drivable area (e.g., on a pavement).
• “next_intersection” indicates the vehicle’s intended action at the forthcoming intersection. Possible values are ← (turning left), → (turning right), *↑* (continuing straight), and Ø if no upcoming intersection is present.
• “objects_nearby” indicates the presence of potentially influential objects detected by the vehicle’s front camera.
• “stop_area_nearby” indicates the presence of an area requiring the vehicle to stop or yield. Possible values are stop (stop sign), yield (yield sign), TurnStop (areas requiring the vehicle to yield to oncoming traffic when executing a turn), or Ø if no relevant stop area is detected.
• “crosswalk_nearby” indicates the presence of a crosswalk within the vehicle’s frontal area.
• “traffic_light_nearby” indicates the presence of a traffic light facing the vehicle within its frontal area, regardless of the traffic light’s color status, as this information is not available in the dataset.
• “pedestrian_nearby” indicates whether the vehicle’s front camera detects any close pedestrians.
• “two_wheel_nearby” indicates whether any two-wheeled road user (e.g., cyclists or motorbikes) is detected by the front camera.
• “block_progress” represents the vehicle’s progression within the current lane block, with values specifying whether the vehicle is at the beginning, middle, or end of the lane. Ø is assigned if the vehicle is outside the drivable area or if its orientation relative to the lane could not be determined.
• “idle_time” represents how long the vehicle has been idling in the same state.

**Table 6:** Variables used to describe the driving domain by each discretizer

	Variables (domain)
*D*_0*a*_	velocity(stopped, moving), steering(*↑*, ←, →), lane_position(aligned, center, opposite, Ø), next_intersection(*↑*, ←, →, Ø), objects_nearby(yes, no), stop_area_nearby(stop, yield, TurnStop, Ø), crosswalk_nearby(yes, no), traffic_light_nearby(yes, no)
*D*_0*b*_	*D*_0*a*_ \ {velocity(stopped, moving)}, ∪ {velocity(stopped, low, medium, high)}
*D*_1*a*_	*D*_0*a*_ ∪ {pedestrian_nearby(yes, no), two_wheel_nearby(yes, no), block_progress(start, middle, end, Ø)}
*D*_1*b*_	*D*_0*b*_ ∪ {pedestrian_nearby(yes, no), two_wheel_nearby(yes, no), block_progress(start, middle, end, Ø)}
*D*_2*a*_	*D*_1*a*_ ∪ {idle_time(0, 1–4, 5+)}
*D*_2*b*_	*D*_1*b*_ ∪ {idle_time(0, 1–4, 5+)}

The PGs generated by the proposed discretizers are evaluated using static metrics to identify the representation that minimizes uncertainty in action and future-state predictions.

The results are summarized in Table 7. The entropy values are consistent with the theoretical expectations. The action entropy *H*_*a*_ decreases as the discretization becomes more detailed, as finer-grained graphs attenuate the uncertainty about the agent’s following action. Conversely, the world entropy *H*_*w*_ increases with the complexity of the discretizer, reflecting the heightened uncertainty about future states. The lowest overall entropy is achieved with the simplest representations *D*_0*a*_ and *D*_0*b*_.

**Table 7:** Static metrics for the driving agent

*D*	*H*	*H*_*a*_	*H*_*w*_
*D*_0*a*_	**2.54**	1.55	**0.99**
*D*_0*b*_	**2.54**	1.49	1.05
*D*_1*a*_	2.73	1.44	1.29
*D*_1*b*_	2.61	1.35	1.26
*D*_2*a*_	2.74	1.43	1.31
*D*_2*b*_	2.62	**1.34**	1.28

The best values are marked in bold.

#### Desire definitions: Autonomous driving

The reduced temporal horizon of the driving scenes, which are generated by chunking longer trajectories, directs emphasis toward short-term desires (e.g., yielding to a pedestrian) rather than toward longer-term ones (e.g., reaching a destination). We assume that the vehicle operates with the intent of achieving two categories of desires: “safe” and “unsafe” desires.

Safe desires relate to ordinary maneuvers executed for navigating traffic and include the following desires:

• Lane keeping: this state region includes all states where the vehicle is in motion, oriented forward, aligned with the direction of its lane, and manifests no intention to turn at an upcoming intersection. The actions to be performed include accelerating, decelerating, or maintaining the same pace while preserving forward orientation.
• Turn left: this state region includes all states where the vehicle steers toward the left at the end of a road block, with the intention to execute a left turn at the forthcoming intersection. The actions to be performed include turning left, which may be combined with acceleration or deceleration. The definition applies analogously for the desire to turn right.
• Lane change (to lf): this state region includes all states where the vehicle is in motion, oriented toward the left, and located on a lane or road divider. The actions to be performed include turning left, possibly combined with acceleration or deceleration, or proceeding straight (in the case of gradual lane changes). The definition applies analogously for the lane change (to rt) desire.
• Approach traffic light: this state region includes all states where the vehicle is traveling in the proximity of a traffic light and possible actions involve halting and deceleration.
• Approach stop sign: this state region includes all states where the vehicle is in motion in the proximity of a stop sign and far from traffic lights that could influence its behavior. Desirable actions include halting and deceleration.
• Peds at crosswalk: this state region includes all states where the vehicle is traveling in the proximity of a crosswalk and detects one or more pedestrians in the front. Desirable actions include halting and deceleration.
• Jaywalking peds: this desire is analogous to peds at crosswalk, with the distinction that the pedestrians are located away from a designated crosswalk (e.g., jaywalking individuals or construction personnel within the roadway).

Unsafe desires, by contrast, represent reckless or non-compliant driving behaviors and are fulfilled when actions deemed hazardous are executed within the corresponding desirable state regions. This category is introduced to support the identification of dangerous driving tendencies and includes the following desires:

• Ignore two-wheel vehicle: this state region corresponds to the set of states where the vehicle is traveling oriented forward, at high velocity, and detects two-wheeled vehicles ahead, and possible actions involve acceleration.
• Ignore peds (high): this state region includes all states where the vehicle is traveling at medium or high velocity while detecting one or more pedestrians ahead, and possible actions are those that do not entail deceleration.
• Ignore peds (low): this state region includes all states where the vehicle is driving at a low speed. It detects one or more pedestrians ahead, and possible actions are those that entail acceleration.
• Ignore stop sign: this state region is equivalent to that of approach stop sign; however, the set of actions excludes deceleration or stopping maneuvers.
• Out of driving area: this state region includes all states where the vehicle is located outside of the drivable area. This desire is defined as an achievement goal, and no specific action is required to fulfill it.

#### Intention metrics: Autonomous driving

**Figure 9:**
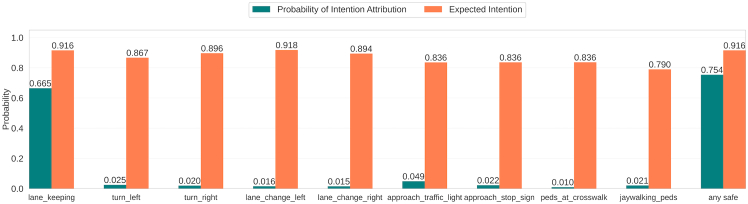
Expected intention and probability of intention attribution for the driving agent using discretizer *D*_1*b*_ (safe desires)

**Figure 10:**
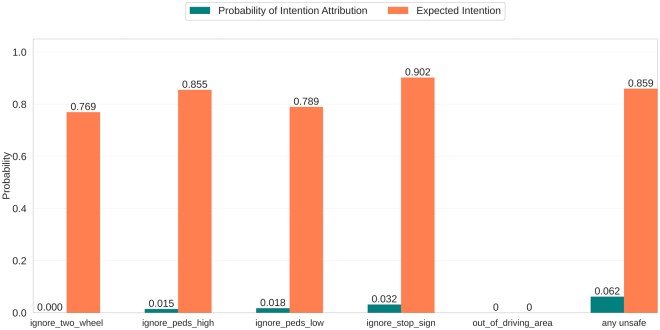
Expected intention and probability of intention attribution for the driving agent using discretizer *D*_1*b*_ (unsafe desires)

Figures 9 and 10 show intention metrics for the driving agent, with a 0.5 commitment threshold. The analysis focuses on the discretizer *D*_1*b*_. Most scenes in the dataset involve straightforward driving, with a limited range of complicated traffic situations and hazardous maneuvers. The nature of the dataset is reflected in the intention metrics: lane keeping has the highest intention probability, indicating a strong association between this intention and the agent’s behavior. In contrast, the metric is low for the remaining desires.

The overall high expected intention probability of the desires corroborates the reliability of the ascribed intentions. An exception is the out of driving area desire, with zero values for both metrics, suggesting that the driver never intends to travel outside the carriageway.

The metrics in Figure 9 illustrate a high interpretability and reliability of the agent’s intentions for safe desires: 75% of the time, we can attribute a safe intention to the agent, which gets fulfilled 92% of the time.

Although the expected intention values are high, they are not absolute. This allows us to point out the portion of behavior where an intention is ascribed but not fulfilled, which is critical for some desires. For example, in the case of approach traffic light, the intention is fulfilled most of the time (84%); however, in the remaining instances, the vehicle does not slow down or stop, possibly due to the presence of a green traffic light (information not available in the dataset).

Similarly, for stop signs and pedestrian-related desires, there are cases where the driver holds the intention to fulfill the desire but does not slow down or stop when being in the desirable state region. This behavior is further examined through the analysis of unsafe desires below.

The metrics presented in Figure 10 reveal that the intention to fulfill any unsafe desire is rarely attributed (6% of the time); nevertheless, when attribution does occur, these intentions are fulfilled in most cases (86%). As the dataset lacks instances of extreme traffic violations (e.g., pedestrian or cyclist collisions), we hypothesize that when desires related to pedestrians and two-wheelers are ascribed, the driving agent is most likely executing hazardous avoidance maneuvers around these vulnerable traffic participants.

The ignore stop sign desire emerges as the most frequently attributed unsafe desire, with the highest reliability of fulfillment, substantiating the agent’s driving tendency to approach stop signs without braking or stopping.

Considering both safe and unsafe desires, *any* intention can be attributed to the agent’s behavior 76% of the time, and in 92% of these cases, the associated desire is fulfilled, substantiating the high interpretability of the driver’s behavior and the reliability of the resulting explanations.

**Figure 11:**
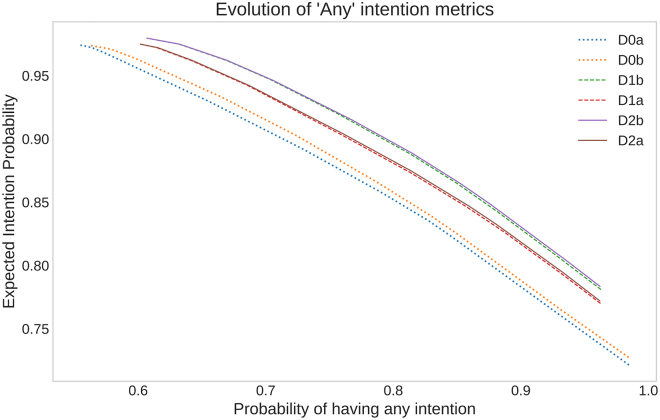
Probability of intention attribution (interpretability) and expected intention probability (reliability) progression as the commitment threshold changes for all six discretizers of the driving agent

Figure 11 shows the progression of the probability of intention attribution and the expected intention as the commitment threshold varies for *any* desire (safe and unsafe) across all discretizers. The high intention metrics observed indicate that the hypothesized desires provide a satisfying interpretability-reliability trade-off across all discretizers. *D*_1*b*_ and *D*_2*b*_ achieve the highest area under the curve, making them the most suitable representations for ascribing intentions to the agent’s behavior (*D*_1*b*_ is a more efficient choice due to its reduced state space). Note that the best discretizations according to intention metrics (*D*_1*b*_ and *D*_2*b*_) differ from those identified as optimal by static metrics in Table 7, where simpler representations *D*_0*a*_ and *D*_0*b*_ are favored. The final choice depends on the priority of the explainee: if certainty in predicting actions and future states is more critical, *D*_0*a*_ or *D*_0*b*_ is preferable; if attributing intentions to the driving agent is the main focus, then *D*_1*b*_ or *D*_2*b*_ should be selected.

#### Revision pipeline example: Autonomous driving

**Figure 12:**
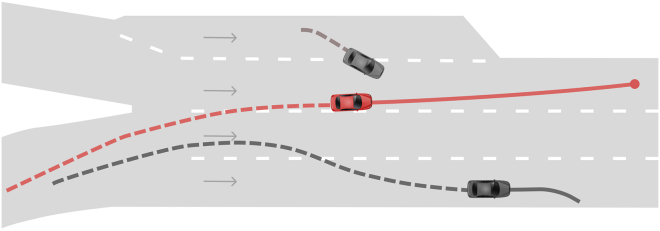
Illustration of scene 1,084 of the dataset showing the driving agent in red, with its final state marked by a red dot, and surrounding vehicles in gray The driving agent merges from a secondary road onto the main road, transitioning to the middle-left lane. While concluding the lane-change maneuver, a vehicle merges from the far-left lane without signaling, causing the agent to brake sharply. The second vehicle decelerates as well and interrupts its merging attempt. The agent then completes the lane change and proceeds straight. The position of the vehicles in the visualization is acquired at state *s* = 20, when the agent is nearly halted due to the abrupt intrusion of the other vehicle.

We apply the revision pipeline to examine a real-world driving scene from the dataset and compare it with the corresponding ascribed intentions. The scene, available at https://www.nuscenes.org/nuscenes?sceneId=scene-1084, is described in Figure 12.

**Figure 13:**
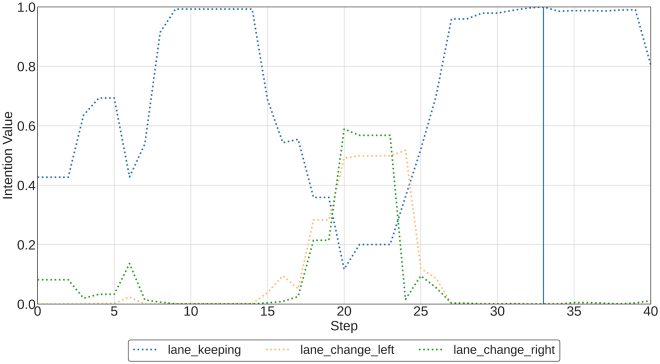
Progression of intentions during a driving scene Intention values at each timestep are marked with dotted lines and desire completion with vertical solid lines. Each color represents a desire: blue for lane keeping, orange for lane change (to lf), and green for lane change (to rt). For a more intelligible visualization, only desires that attain an intention value greater than 0.2 at least once throughout the scene are shown.

Figure 13 shows the temporal evolution of the agent’s intentions throughout the scene. In the first region (time steps 0–14), the agent exhibits an increasing commitment to lane keeping, with a minor decrease observed at time step 6, and remains extremely close to fulfilling its desire. The temporary drop may be attributed to the realignment move after the curve, which merges onto the main road.

In the second region (time steps 15–24), the agent’s commitment to lane keeping decreases. The agent initially forms an intention to execute a lane change to the left lane; however, this is promptly overtaken by a (competing) intention to change to the right lane. This reorientation can be attributed to the sudden pulling out of a second vehicle from the roadside and the driving agent’s aim to avoid a collision.

Nevertheless, both lane change desires do not reach high intention values and remain unfulfilled, which reveals an anomalous behavior and may suggest the presence of a hidden, higher-priority desire (e.g., the desire to avoid collisions).

In the third region (time steps 25–40), the vehicle restores and fulfills its intention of lane keeping, in line with the real scene.

A final comparison between the driving scene and the temporal evolution of intentions highlights an initial discrepancy between the observed behavior and the ascribed intentions. In the original scene, the agent starts executing a leftward lane change shortly after merging onto the main road, whereas this intention is ascribed later in time.

Further analysis reveals that this anomaly is attributed to an inconsistency in the nuScenes map: the initial segment of the lane dividers (the white dashed lines in Figure 12) is not depicted on the map, despite being present in the real-world environment.

This omission affected the discretization of the agent states throughout the scene; specifically, the predicate lane_position remained classified as aligned for longer than expected before switching to center, which resulted in a minimal but noticeable misalignment in the computed intentions.

## Discussion

Our approach to IPGs offers a framework for enhancing explainability in opaque agents. However, the effectiveness of our method is inherently dependent on the quality of state discretization and the ability to infer meaningful desires. Also, the construction of a PG imposes additional requirements on the explainee.

One obvious requirement is the necessity of outer desires: as part of the process, the explainee must provide formal descriptions of what constitutes desirable behavior. When attempting to infer an agent’s desires from statistics alone (e.g., by using notions of criticality or low entropy), spurious correlations may yield nonsensical explanations or distort the method’s value. Moreover, desirable actions discovered automatically burden the explainee with the task of determining why those should indeed be classified as desires. When explicitly provided, the reasons for desirability are apparent to the explainee (since they already believed the behavior to be desirable). Thus, they only need to be tested via the proposed pipeline. As such, we strongly discourage IPG users and system designers from fully automating desire discovery: at a minimum, it is necessary to communicate the rationale of the selected desires to explainees. Otherwise, intentions of that desire become no better than noise and can even mislead explainees, who create a wrongful understanding of why a predicate combination is desirable.

Another key challenge lies in defining appropriate state representations in the exposed examples: predicates. We have found that predicate-based descriptions strike a balance between interpretability and computational feasibility. Beyond computational and data requirements, the discretization is performed so that state descriptions are shared between the explainee and the explainer. These descriptions are necessary when performing the original types of explanations^20^ as well as the how question. However, finding a way to discretize environments can be challenging in complex environments (such as those with image input). Even with optimal automatic discretization,^98^ environments with large, complex state spaces, such as chess, will lose essential information when providing explanations. These environments remain as future work for PGs. Future work could explore automated techniques for state abstraction and dynamic discretization.

Additionally, while our method provides insights into agents’ intentions, it does not inherently resolve issues related to explainability in MA or adversarial settings. Further research is needed to extend these principles to more complex environments with multiple interacting agents.

The robustness of the metrics stems from two necessary conditions: good estimators for the policy and world model and a strong definition within the formalism of what the metrics measure. The estimation condition is the limiting factor when switching domains, as obtaining good estimators of *P*(*s*′, *a*|*s*) can be challenging and currently depends on human feedback. This is why we present multiple static metrics to assess whether the distribution is representative and to detect limitations of the technique, particularly with sparse state spaces. We would like to note that practitioners may be tempted to estimate *P*(*s*′|*a*, *s*) instead of computing it from a dataset. This is feasible and could outperform the frequentist method presented for PG. However, in adding a (potentially opaque) model as the source of these beliefs, the need for explainability is shifted rather than resolved, as explainees may now ask, “Why did you believe *P*(*s*′|*a*, *s*) = *x*?” With the frequentist approach, the answer is epistemically solid (observation-wise, it happened). With an opaque model, it becomes a new problem instead.

The presence of poor results in some experiments leads to an important acknowledgment: IPGs cannot magically make a bad agent explainable. Moreover, agent practitioners with a background in verification or symbolic agents may think that there is no way to infer agent intention or other properties for RL agents and that they cannot be said to be rational. While this paper has not directly studied it, we believe methodologies such as the ones presented here may offer a way to validate these claims or find a pragmatic soft definition of rationality through understanding how frequently and how reliably an agent pursues goals, helping a practitioner decide when RL agents are a valid option for a domain or use case. Additionally, should RL agents be too opaque to deploy in some environments, ongoing work^85^ presents the possibility of substituting them with PG surrogates. With the addition of intentions, this research line may be better informed about how the surrogate agent behaves, accounting not only for the reward signal but also for desires.

Finally, the reliance on human-defined desires introduces potential subjectivity. Although we propose metrics to quantify interpretability and reliability, validating these against human cognitive models remains an open research direction. Future work may incorporate learning-based methods to automatically refine intention predictions and mitigate biases introduced by manually defined desires. The trade-off between wanting to receive intention explanations and ensuring correctness is crucial: we quantify this by assessing the probability that an intention is assigned and the likelihood that the agent fulfills it.

Additionally, our choice of PGMs enables several key contributions in our work. Specifically, PGMs provide a structured approach to modeling agent intentions by incorporating both state transitions and probabilistic dependencies. This formalism allows us to define intention-aware metrics, support a revision pipeline for refining state representations, and enhance explainability through structured causal reasoning. The ability to infer agent goals from partial observations—without requiring access to internal states or reward functions—is a direct consequence of this choice. Furthermore, PGMs facilitate a trade-off between model interpretability and expressiveness, making them particularly well suited for environments where state abstraction is necessary.

Nonetheless, we acknowledge that different formalisms beyond PGMs could also be suitable for modeling agent intentions. As mentioned, PGMs provide a strong theoretical foundation, but alternatives may offer distinct advantages in specific contexts. In particular, expanding our approach to problems with highly complex, sparse state spaces (e.g., chess) would require modifications to state discretization techniques.

By selecting agents with varying degrees of rationality relative to the environment’s reward function, we can examine how interpretability correlates with different behavioral strategies. Agents that closely align with the reward function exhibit more predictable, goal-directed behavior, while others display deviations that can challenge traditional interpretability techniques. This enables us to assess the robustness of our intentionality metrics across a broad range of agent decision-making paradigms. Thus, our findings suggest an interesting relationship between agent rationality and explainability. Our method is based on desires and goals, which are tied to agent rationality. Thus, examining how different degrees of rationality affect interpretability is an opportunity for future, more in-depth study. In addition, we highlight the potential for real-world applications such as modeling human driving behavior, where preliminary results indicate promising directions for future work. As an outcome of this process, we are optimistic about using this method in applications alongside human explainability. One of the key contributions of this paper is that, by using the method proposed, there is a way to automatically create policies for easily understandable agents that mimic the behavior of an original agent, thus enabling our method as a theory-of-mind model for understanding the behavior of others in MA systems. In addition, the availability of state intentions may be useful for better designing rewards for RL agents (e.g., by locating sparse regions and populating them to go toward intention-attributed regions) or improving other types of agent implementations. Finally, we believe the insights provided in this paper about the necessity of having a world model (i.e., *P*(*s*′|*a*, *s*)) and how it enables teleological explanations will be key in designing transparent agents. The introduction of such models may also help the RL community.^99^

### Concluding summary

The proposed framework allows for attributing intentions and extending IPG models, which enable teleological explanations with the flexibility to allow a human explainee to modify the technique and content of the explanations to suit their needs. The encoded information of desires provides new types of explanations, such as “What do you intend to do now?”, “How do you plan to do it?”, and “For what purpose did you take this action now?”, in a concise manner, directly related to the content of the question, and enabling the explainee to ask further questions based on the answers to previous ones. In addition, the PG model is instrumented with metrics to evaluate the reliability and interpretability of the behavior, and the trade-off is made explicit with the introduction of a designer-defined parameter: the commitment threshold. All of these properties are aligned with Grice’s maxims of communication (quantity, relation, quality, and manner).

Our approach builds upon IPGs while incorporating a structured pipeline for intention inference and evaluation. Our key contributions can be summarized as follows:

• We propose an iterative workflow for constructing an IPG from observed agents, integrating human-provided desires with probabilistic state transitions. We notice that our results were good out of the box, with the method being resistant to changes in the discretizer; however, in front of a different explainee, the method can be modified if these results are not agreeable.
• Our proposed framework has only two computationally costly processes: obtaining agent observations and running intention propagation. The pipeline is designed so that these can be run sparingly and that computation can be reused in most circumstances.
• We employ a set of quantitative metrics to assess the interpretability and reliability of agent explanations. We add explanations on how each metric should be interpreted and how they can be used to improve the IPG*.*
• We show a qualitative example of a downstream task, showcasing how an IPG can be used to debug a system, and suggest how it can serve to control and improve the agents being explained as well as the IPG itself.
• We validate our approach using two use cases: Overcooked-AI, which is a simpler environment but illustrates an MA system with some agents trained with RL and some via imitation learning, and the nuScenes dataset for AD, a much more complex environment where the agent is a human driving in the real world. This demonstrates that the method is truly model agnostic and can work with opaque models. This presents an optimistic perspective on the model’s scalability.

Although this process requires external knowledge and is not off-the-shelf, the provided heuristics, as well as the revision pipeline, enable guided iteration in the modeling by naturally gathering and exposing its shortcomings. We believe that the whole proposed methodology can be applied to many tasks (Figure 1). In the following sections, we discuss current limitations and how to address them.

### Limitations

While the method is demonstrated to work in two very different environments, there are several limitations regarding when it can be applied and whether the method is useful. The former is generally tied to scale and efficiency of the method, while the second is related to the practical utility and what particular problems an IPG solves. We discuss avenues for solving them in future work.

From an efficiency perspective, there are significant limitations. The method for obtaining the PG requires observing the agent for an extended period to ensure reliability. While this can be the case for agents made by the PG designer or those that the PG has frequent access to, the method does not work with infrequently available agents or those that frequently modify their own behavior, as the agent drifts from the PG probabilities.

Furthermore, the cost of computing intentions can be very high in PGs with high-probability loops. This can be seen in the AD use case: many states are such that executing actions (e.g., continue forward) maintains the current state. This increases the computational cost substantially. It should be possible to use analytical methods to automate and precisely compute the increase in intention in loops, but that remains future work.

Finally, in terms of efficiency, numerous complex state spaces can be abstracted using discretizers and work for many use cases (as can be seen from the use of an IPG for driving). However, there are environments where any abstraction or aggregation can remove crucial details needed to understand the environment. For example, chess, while it has a discrete state space, has such state sparsity that building a PG would require a discretizer to group states of similar characteristics and, in the process, lose information that is needed to properly explain chess, making IPGs an ill-suited technique without substantial modification.

From the perspective of applicability, the IPGs introduced in this paper are evaluated for trustworthiness and reliability, primarily yielding limited quantitative results on the effectiveness and extent of increased trust in explainees. The explanation algorithms introduced are examples of what sort of information an IPG can provide to build explanations. The reason we do this is 2-fold: in background, we showed that PGs have methods to produce explanations in natural language, and we also remark on the emphasis on making *trustworthy* (i.e., truthful and faithful) explanations before attempting to make them *trust-increasing* ones. As such, we have deferred making user studies to future work.

Along similar lines, we have shown that the intentions computed in this paper can be used for other downstream tasks, such as debugging the agent or reporting situations in which the agent behaves unexpectedly. While the suggested applications are feasible, the evaluation shown is purely qualitative; automating and performing a quantitative evaluation of how IPGs can be used to improve model performance and for other downstream tasks is outside the scope of this paper.

Finally, a theoretical limitation of the work is that the agent cannot provide responses to actions or transitions that the PG has not experienced. For example, the agent cannot reply to questions about doing an action *a* in a state *s* if this has never been observed (e.g., the agent never does this). This sort of counterfactual explanation requires more advanced causal concepts (i.e., intervention, using *P*(*s*′|*do*(*a*), *s*) when *P*(*a*|*s*) = 0).

### Future work

While this paper has demonstrated the feasibility of applying IPGs to a wide range of use cases, several limitations to the model’s usefulness remain. To address this, in future contributions, we intend to do the following.

• Improve the explanation algorithms in terms of answer format and visualization, then run user studies to show their impact on explainee trust. In particular, the how question may benefit from filtering more confusing predicates out of the response and being in a visual (as opposed to textual) format.
• Leveraging the ancillary models present in the agent to reduce data necessities. If the designer has access to the agent policy (as *P*(*a*|*s*) or a similar formalism), it becomes unnecessary to estimate the function. Likewise, if the agent has a world model, building a PG becomes much less computationally expensive, and the answers are tied to the model. While this makes the technique not model agnostic, the answer format and the metrics associated with it remain model agnostic, preserving the desiderata introduced in background that explain why these techniques are necessary for establishing baselines for XAI*.*
• Modifying the propagation algorithm for loop detection mechanisms. A high-probability loop in a PG causes many unneeded iterations of the algorithm. Detecting loops and accounting for them when doing intention propagation (via analytical methods) should drastically reduce the computation time of intention propagation for more complex cases that require it.
• Learning the PG during an agent’s training (in cases where an agent is learning or modifying its policy, mainly RL). The most computationally expensive part to estimate in a PG is transition information (i.e., *P*(*s*′|*a*, *s*)), which is independent of the policy. As such, to increase data efficiency, the training process can be parallelized to estimate transition information.
• Furthermore, because the policy influences which states an agent visits, learning the PG from a fixed agent is more likely to yield less information about state transitions due to the reduced variety of states explored. This also helps address the issue of counterfactual explanations, as even if the agent no longer performs a particular action in a state, information from training remains about the consequences of that action.
• Interventions and counterfactuals. Similarly to building a PG from learning agents, if a designer has access to the environment, they can perform interventions (i.e., modify the agent’s actions) to obtain complementary information for the PG*.* This would effectively allow the IPG to generate more counterfactual questions rather than relying solely on observational data and correlations.
• Tackling undiscretizable environments. In the limitations section, we discussed how sparse environments with a lot of detail scale poorly for IPGs, as simplifications for representing states in the graph also remove crucial data necessary to estimate the policy and transition probabilities. However, the insights in this paper focus on intentions, which are exclusively contingent on the policy and transition probabilities. We argue that it should be possible to export the methods presented in this paper to provide the telic explanations presented here *without* a PG. Instead, it should be possible to estimate the definition of intention given in this paper from other data and use the same algorithms in other, non-PG representations. For instance, the chess environment is a good candidate for this objective.

## Resource availability

### Lead contact

Requests for further information and resources should be directed to and will be fulfilled by the lead contact, Sergio Alvarez-Napagao (sergio.alvarez@bsc.es).

### Materials availability

This study did not generate new materials.

### Data and code availability

• Code and experimental results generated in this study for the Overcooked-AI experiments are available at GitHub (https://github.com/HPAI-BSC/intention-aware-pgs) and archived in Zenodo.^100^
• Code and experimental results generated in this study for the Overcooked-AI experiments are available at GitHub (https://github.com/HPAI-BSC/ipg4av) and archived in Zenodo.^101^
• The *pgeon* library used for the development of these experiments is available at GitHub (https://github.com/HPAI-BSC/pgeon) and in Zenodo.^96^

## Acknowledgments

This work has been partially supported by the AIXPERT (grant agreement ID: 101214389), AI4CCAM (grant agreement ID: 101076911), and HumanE-AI-Net (grant agreement ID: 952026) European projects and fellowships to V.G.-A., A.T., and S.M. within the “Generación D” initiative, Red.es, MTDFP, for talent attraction (C005/24-ED CV1). This work was partially funded by the European Union NextGenerationEU funds, through PRTR.

## Author contributions

Conceptualization, V.G.-A., S.A.-N., and A.T.; methodology, V.G.-A.; software, V.G.-A., S.A.-N., A.T., and S.M.; validation, V.G.-A., S.A.-N., A.T., and S.M.; formal analysis, V.G.-A. and J.V.-S.; investigation, V.G.-A., A.T., and S.M.; resources, V.G.-A., A.T., and S.M.; data curation, V.G.-A. and A.T.; writing – original draft, V.G.-A.; writing – review & editing, V.G.-A., S.A.-N., A.T., S.M., U.C., and J.V.-S.; visualization, V.G.-A. and S.M.; supervision, S.A.-N., U.C., and J.V.-S.; project administration, S.A.-N. and U.C.; funding acquisition, S.A.-N. and U.C.

## Declaration of interests

The authors declare no competing interests.
